# The Sorcerer II Global Ocean Sampling Expedition: Metagenomic Characterization of Viruses within Aquatic Microbial Samples

**DOI:** 10.1371/journal.pone.0001456

**Published:** 2008-01-23

**Authors:** Shannon J. Williamson, Douglas B. Rusch, Shibu Yooseph, Aaron L. Halpern, Karla B. Heidelberg, John I. Glass, Cynthia Andrews-Pfannkoch, Douglas Fadrosh, Christopher S. Miller, Granger Sutton, Marvin Frazier, J. Craig Venter

**Affiliations:** 1 J. Craig Venter Institute, Rockville, Maryland, United States of America; 2 University of Southern California, Los Angeles, California, United States of America; 3 Molecular Biology Institute, University of California at Los Angeles, Los Angeles, California, United States of America; University of Liverpool, United Kingdom

## Abstract

Viruses are the most abundant biological entities on our planet. Interactions between viruses and their hosts impact several important biological processes in the world's oceans such as horizontal gene transfer, microbial diversity and biogeochemical cycling. Interrogation of microbial metagenomic sequence data collected as part of the Sorcerer II Global Ocean Expedition (GOS) revealed a high abundance of viral sequences, representing approximately 3% of the total predicted proteins. Cluster analyses of the viral sequences revealed hundreds to thousands of viral genes encoding various metabolic and cellular functions. Quantitative analyses of viral genes of host origin performed on the viral fraction of aquatic samples confirmed the viral nature of these sequences and suggested that significant portions of aquatic viral communities behave as reservoirs of such genetic material. Distributional and phylogenetic analyses of these host-derived viral sequences also suggested that viral acquisition of environmentally relevant genes of host origin is a more abundant and widespread phenomenon than previously appreciated. The predominant viral sequences identified within microbial fractions originated from tailed bacteriophages and exhibited varying global distributions according to viral family. Recruitment of GOS viral sequence fragments against 27 complete aquatic viral genomes revealed that only one reference bacteriophage genome was highly abundant and was closely related, but not identical, to the cyanomyovirus P-SSM4. The co-distribution across all sampling sites of P-SSM4-like sequences with the dominant ecotype of its host, *Prochlorococcus* supports the classification of the viral sequences as P-SSM4-like and suggests that this virus may influence the abundance, distribution and diversity of one of the most dominant components of picophytoplankton in oligotrophic oceans. In summary, the abundance and broad geographical distribution of viral sequences within microbial fractions, the prevalence of genes among viral sequences that encode microbial physiological function and their distinct phylogenetic distribution lend strong support to the notion that viral-mediated gene acquisition is a common and ongoing mechanism for generating microbial diversity in the marine environment.

## Introduction

Viruses comprise the smallest and most abundant biological agents within the entire biosphere. Our world's oceans are teeming with viruses, with approximately 10^7^ ml^−1^ of surface seawater [1]. Bacteriophages, or viruses that specifically infect bacteria, are the numerically dominant type of virus in the marine ecosystems; often outnumbering their hosts by at least one order of magnitude [2]. Currently, viral infection and subsequent lysis of host cells is viewed as the most efficient means of transformation of microbial biomass into dissolved organic matter (DOM) thereby disrupting the biological pump [2]–[6]. Furthermore, phages are recognized as important mediators of horizontal gene transfer, influencing the diversification and evolution of bacterial lineages [7]–[10]. Both virulent and temperate phages are known to facilitate the transfer of genes from one host to another [2], [11], yet temperate phages have the ability to establish silent infections with their hosts through genomic integration [12]. Once integrated into a host's genetic material (either chromosome or plasmid), prophages impact their hosts on multiple levels; from lysogenic conversion (the expression of phage-encoded genes) to strain diversity [13], [14].

Cyanophages, viruses that specifically infect cyanobacteria, are abundant components of surface marine bacteriophage communities [15]–[18]. Similar to phages that infect heterotrophic bacteria, cyanophages can impact the diversity of cyanobacterial communities by mediating bacterial mortality, horizontal gene transfer and potentially lysogenic conversion [19], [20]. Several studies have demonstrated that the marine picocyanobacteria *Prochlorococcus* and *Synechococcus*, both extremely abundant in the surface waters of the world's oceans [21], [22], are highly susceptible to infection by cyanophage [17]–[19], [23], [24]. As these two members of the cyanobacteria are important contributors to photosynthesis in oceanic waters [21], [22], [25], [26], there have been a number of investigations aimed at understanding the genomic contents of their phages. Sequencing of *Prochlorococcus* and *Synechococcus* phage genomes has led to exciting new revelations about the extent of lateral gene transfer between viruses and their hosts. For instance, certain genes involved in host metabolic functions, such as those involved in photosynthesis, have been acquired and retained by viruses [27]–[32]. The existence of such genes within phage genomes makes them available for subsequent transfer back to their hosts and to other viruses, expanding the overall size of the gene pool and influencing the evolution of both viruses and their hosts [30], [33].

A better understanding of the contribution of viral genomes to microbial environmental processes is just starting to be revealed through the application of metagenomic techniques. The majority of viral metagenomic studies to date have primarily focused on DNA isolated from material passing through filters <0.22 µm in size, the fraction that contains the bulk of virus-like particles. [34]–[36]. These targeted viral metagenomic investigations revealed that viral communities are extraordinarily diverse on both local and global scales [34]–[36]. Furthermore, the analysis of marine viromes across four oceanic regions suggests that viral community composition and nucleic acid type (i.e. dsDNA vs. ssDNA) is a function of geographic location and that vastly different environments support similar viral communities that differ only in the abundance of the dominant viral members [36]. In contrast to these targeted investigations, Delong and colleagues reported on marine virus-host interactions along a vertical transect of the North Pacific Subtropical Gyre by adopting a community-wide metagenomics approach [37]. The viral sequences analyzed in this study originated from the microbial fraction (0.22 µm-1.6 µm) of community DNA rather than from purified viral particles [37]. Analysis of fosmid end-sequences indicated that in the waters surrounding Hawaii, the highest proportion of viral sequences originated from the photic zone, were predominantly cyanophage, and decreased precipitously with depth [37]. Furthermore, a small proportion of cyanophage-related sequences (11%) in the photic zone appeared to be viral versions of genes involved in various host-specific metabolic functions [37].

Here we report the results of our analysis of viral dsDNA sequences recovered from the microbial fraction (0.1 µm–0.8 µm) of 37 new surface marine, freshwater and hypersaline samples collected during the first phase of the Sorcerer II Global Ocean Sampling (GOS) Expedition [38], [39] with additional data from the four stations sampled as part of the Sargasso Sea pilot study [40]. In this paper, we sought to characterize the viral sequences with respect to their occurrence and distribution across a diverse range of aquatic ecosystems. We used comparative genomic analyses to functionally characterize viral sequences through sequence similarity clustering and to elucidate the importance of viral acquisition of host genes encoding for environmentally significant metabolic functions in aquatic environments.

## Results

A total of 37 marine surface water samples were collected between August 8, 2003 and May 22, 2004 during the first six legs of an oceanographic expedition; originating in Halifax, Nova Scotia and ending in French Polynesia. In addition to mostly marine surface water, a few samples were collected from distinctly different aquatic and terrestrial environments such as a freshwater lake, a warm marine seep, a coastal mangrove forest and a hypersaline lagoon. Table S1 contains a description of the sampling sites and a subset of their accompanying physical-chemical data. Additional sample details are described in Table 1 of Rusch et al. (2007) [41]. We combined the sequence data that was generated from the 37 new samples with additional data collected from four stations that were part of the Sargasso Sea pilot study [42]. Although the original dataset that was generated from the Sargasso Sea pilot study was examined for the presence of viruses, these investigations were restricted to bacteriophage [40]. In this study, we extended the original analyses to include all viruses that would be captured by the cloning methods employed. Approximately 7.7 million sequencing reads (6.3 billion bp) were produced from the first phase of the GOS expedition. Assembly was conducted with the Celera Assembler using stringent parameters to reduce chimerism and provide a high fidelity consensus sequence. Open reading frames (ORFs) were predicted on the assembled data [41] and scaffolds were given taxonomic assignments according to a BLAST-based voting scheme (see materials and methods).

**Table 1:** Results of qPCR analyses of viral genes of host origin within the viral fraction of aquatic samples.

Host-derived viral subgroup	GS19+GS51 GS20* GS26 GS34			
	Average copy number L^−1^			
petE_4	NA	NA	1.8×10^6^	4.0×10^7^
speD_1	NA	3.8×10^4^	NA	1.4×10^2^
speD_2	1.9×10^6^	NA	1.8×10^6^	1.4×10^5^
speD_3	NA	6.4×10^4^	NA	NA
speD_4	2.6×10^4^	NA	NA	NA
pstS_1	1.2×10^6^	NA	NA	NA
phoH_5	NA	5.6×10^3^	NA	3.9×10^2^
phoH_6	NA	NA	1.3×10^3^	NA
talC_4	5.5×10^4^	NA	1.1×10^4^	4.0×10^3^
talC_7	1.6×10^4^	9.7×10^2^	NA	1.3×10^4^
talC_8	3.0×10^6^	NA	3.4×10^6^	3.1×10^5^
talC_9	4.1×10^4^	7.5×10^4^	3.8×10^4^	4.7×10^4^
talC_12	9.1×10^3^	NA	1.9×10^3^	2.5×10^3^

NA = No detectable amplification

* = Freshwater sample

### Identification of Viral Sequences

A conservative approach was taken in order to distinguish sequences of potential viral origin, i.e. those originating from autonomous viral particles or viable and/or remnant prophages, from the overwhelming majority of microbial sequences. The metagenomic data generated from the microbial fraction of water samples was examined for the presence of viral sequences by comparison to the NCBI non-redundant (nr) protein database. A sequence was given a viral assignment only if the top BLAST homolog was a protein whose origin was clearly attributed to a virus. This analysis resulted in the identification 154,662 viral peptide sequences, 79.3% of which belonged to a multiple sequence assembly scaffolds that were taxonomically assigned as viral. Viral sequences having only their top BLAST hit (E-value≤1e-10) to known viral proteins (the minimum criterion for viral sequence definition) comprised the largest percentage of viral sequences (53.6%), while sequences that had their top 4, 3 and 2 BLAST hits to known viral sequences were 26.1%, 7.0% and 13.2% of the total respectively. Based on this criterion, viral sequences represented approximately 3% of the predicted proteins contained within the GOS microbial dataset [39]. Taking into account that viral genes predicted within metagenomic data and cultured bacteriophage genomes have been shown to share a greater degree of similarity with bacterial rather than viral genes [43], [44],we believe that the number of viral sequences identified within the microbial size fraction is largely underestimated. This is due to the conservative method used to separate the mixture of microbial and viral data and to the relatively limited representation of marine phage genomes in the public sequence databases.

### Classification of viral sequences through protein clustering

We used a sequence similarity-based clustering of proteins from the GOS dataset and previous public protein datasets to assign putative protein function, explore diversity of protein families and measure the degree of novelty added by the GOS sequences [39]. Included in this analysis were the 154,662 viral peptide sequences described here. Of these, 117,123 (76%) fell within 380 protein clusters containing at least twenty sequences. The remaining 24% of sequences were contained within clusters with less than twenty members. The average cluster contained 258 peptide sequences and clusters ranged in size from 20 to 3,020 peptides. Protein cluster membership was useful to assess the accuracy and potential function of viral sequences described above and to also understand the evolutionary history of these proteins.

**Figure 1:**
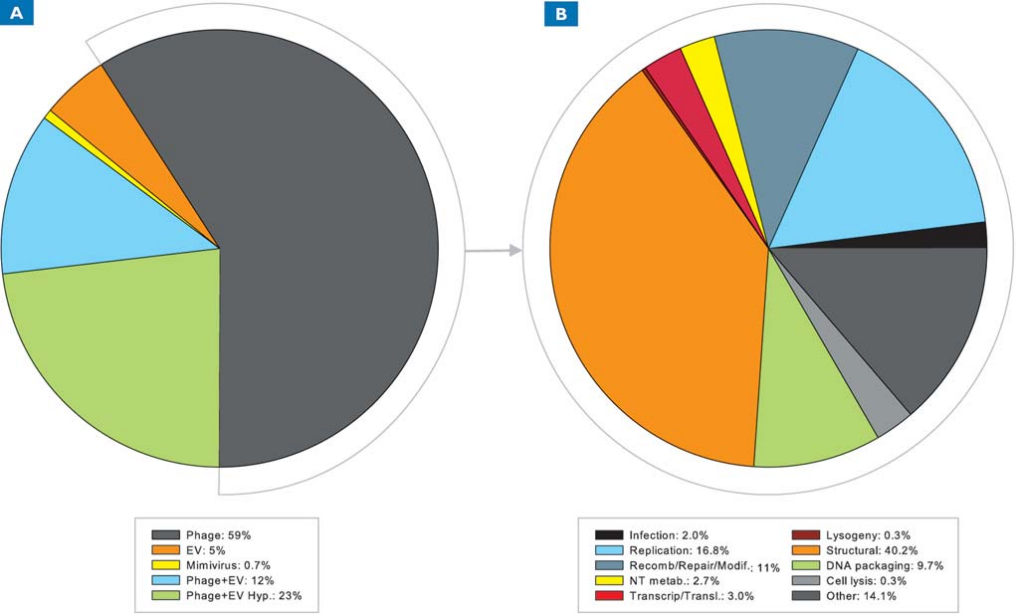
Breakdown of clustered GOS viral sequences by virus type (A) and functional classification of clustered bacteriophage sequences (B). EV stands for eukaryotic virus and Hyp stands for hypothetical.

We only considered viral sequences from the GOS dataset that had top BLAST hits to known viral proteins, although many exceeded this criterion (i.e. had multiple BLAST hits to known viruses). The great majority of clustered viral sequences were most similar to known bacteriophage, with smaller representation from eukaryotic viruses (89% total) (Fig. 1A) (Tables S2, S3, S4, S5), indicating that the majority of sequences identified here as viral were clustered in a fashion consistent with this taxonomic assignment. A small proportion of the clusters containing GOS viral peptide sequences contained either protein sequences that were annotated as either bacterial or eukaryotic, but contained no publicly available viral sequences (2%); or proteins with no similarity to public databases at all (“GOS-only” sequences; 9%) [39]. The fact that a small proportion of GOS viral peptide sequences were not placed in the same clusters as their nearest BLAST neighbors in NCBI nr is most likely a consequence of the specific parameters used in clustering, which only considered similarities that spanned a large portion of the sequence [39]. Therefore, if a public viral BLAST neighbor did not meet the length-based threshold for a match, it was not recruited to the cluster along with the GOS sequences.

For clusters containing viral homologs, the largest proportion of sequences (59%) were exclusively similar to phage proteins, including those involved in the processes of infection, DNA replication, recombination and repair, DNA modification, nucleotide metablolism, transcripton, translation, lysogeny, synthesis of structural proteins, and cell lysis (Fig. 1B). Based on similarity to known prophage sequences, 7% of all viral sequences appeared to be prophage-related. In particular, the clusters containing prophage integrases included162 sequences and represented 0.3% of the clustered viral sequences.

### Detection of environmentally significant viral genes of host origin

As agents of lateral gene transfer, viruses can acquire portions of DNA from their hosts. On occasion, these host-derived genes provide fitness enhancing benefits as has been suggested for the cyanophage versions of the photosynthesis genes *psbA* and *psbD* [27]–[32]. Analysis of translated GOS viral sequences led to the discovery of clusters that contained hundreds to thousands of viral genes encoding host-specific environmentally significant functions (Table S2).

Specifically, four clusters contained photosynthesis-related (PS) viral sequences including plastocyanin (*petE*), high light inducible proteins (HLIP; *hli*), S-adenosylmethionine decarboxylase (*speD*), D1 (*psbA*) and D2 (*psbD*). An additional cluster contained viral transaldolase (*talC*) sequences, which are implicated in cyanophage-mediated carbon metabolism during the dark cycle of host cells [27], [45]. Two clusters contained viral phosphate stress-related sequences including periplasmic phosphate binding proteins (*pstS*) and phosphate starvation proteins (*phoH*). Several additional clusters contained significant numbers of host derived viral genes that potentially contribute to a variety of cellular processes such as vitamin B12 biosynthesis (*cobS*), host stress response (small heat shock proteins), antibiotic resistance (*prnA*) and nitrogen fixation (*nifU*) (Table S2). While it's tempting to speculate that viruses may directly participate in nitrogen fixation in the marine environment through acquisition of host *nifU* genes, it's premature to do so since microbes that do not have the capability to fix nitrogen can also carry this gene and no sequenced marine viral isolates appear to harbor nifU-like sequences [46].

### Neighbor functional linkage analysis of viral genes of host origin

Neighbor functional linkage analysis was conducted on the clustered viral sequences in order to verify that they were encoded on viral rather than non pro-viral regions of bacterial genomes. We inspected the taxonomic assignments of all ORFs that resided on the same scaffolds as the viral sequences in question and the occurrence of each viral, bacterial, eukaryotic, and archaeal sequence was documented. For the metabolic gene families discussed above, the proportion of viral same-scaffold ORFs ranged from 32% to 92% and the occurrence of same-scaffold viral sequences was statistically significant (P<0.05; see materials and methods for details). For PS-related sequences, viral speD sequences had the largest percentage of same-scaffold viral ORFs (92%), while viral psbD sequences exhibited the lowest percentage of same-scaffold viral ORFs (32%). For phosphate stress-related sequences, viral phoH sequences had a much larger proportion of viral neighbors than viral pstS sequences (90% and 33% respectively) suggesting that acquisition of *phoH* genes may be more beneficial to viruses than acquisition of *pstS* genes. Lastly, viral talC sequences displayed a high occurrence of same-scaffold viral neighbors (67%). The significant occurrence of viral neighbors on the same scaffolds as the host-derived viral genes supports the hypothesis that the sources of these sequences are viruses rather than bacterial.

### Quantitative analysis of viral genes of host origin

Quantitative PCR (qPCR) was applied to DNA extracted from the viral fraction of samples collected from five discreet sampling locations (GS19, GS20, GS26, GS34 and GS51-Table S1) in order to further verify the viral nature of host-derived sequences and to determine their relative abundance in the viral fraction of aquatic samples. Yields of viral DNA from sites GS19 and GS51 were initially too low to successfully perform qPCR experiments and were subsequently pooled. The majority of the viral gene families in question (*psbD*, *petE*, *speD*, *talC, pstS*, and *phoH*) that were observed within the microbial fractions of samples were included in qPCR analyses. Alignments of viral nucleotide sequences (see materials and methods) within each gene family resulted in the formation of multiple within-family subgroups based on sequence divergence. From these, we generated consensus sequences from which primers were designed (Table S6).

Out of the viral gene families investigated, only one (*psbD*) did not exhibit any amplification within the four viral DNA samples tested. However, the presence of other PS-related viral genes (*petE* and *speD*) were confirmed within the viral fraction of samples (Table 1). Viral *petE* genes were highly abundant within the viral fraction of GS34, reaching 4.0×10^7^ copies L^−1^ of surface seawater. All four viral *speD* subgroups were also detected within the viral fraction of at least one and often multiple samples, including freshwater (Table 1). Viral *speD* copy number was generally higher in coastal and open-ocean samples (range = 1.4×10^2^-1.9×10^6^ copies L^−1^) than the freshwater sample (range = 3.8×10^4^-6.4×10^4^ copies L^−1^). The presence of viral genes involved in phosphate metabolism (*phoH* and *pstS*) identified within the microbial fractions was also confirmed within the viral fraction of samples. Two of six viral *phoH* sub-groups and one of three viral *pstS* subgroups were detected in viral DNA samples (Table 1). Viral *pstS* genes were also highly abundant (1.2×10^6^ copies L^−1^) in surface seawater; and were approximately three orders of magnitude greater than viral *phoH* copy numbers in other seawater (range = 3.9×10^2^-1.3×10^3^ L^−1^) and freshwater (5.6×10^3^ L^−1^) samples. Lastly, viral *talC* genes were amplified from both seawater and freshwater samples for five of twelve sub-groups (Table 1) ranging from 1.9×10^3^-3.4×10^6^ copies L^−1^ and 9.7×10^2^-7.5×10^4^ copies L^−1^ respectively. Taken together, the results of qPCR experiments indicate that the host-derived viral genes detected in the microbial fractions of samples are viral in nature and that viral genes encoding for environmentally significant, host-specific functions are incredibly prevalent in aquatic samples. If the average viral abundance of surface seawater samples is between 10^7^ and 10^8 ^particles per liter, then our results suggest that viruses carrying metabolic genes of host origin comprise a significant proportion of the viral community across the samples tested.

### Phylogenetic analyses of viral sequences of host origin

**Figure 2:**
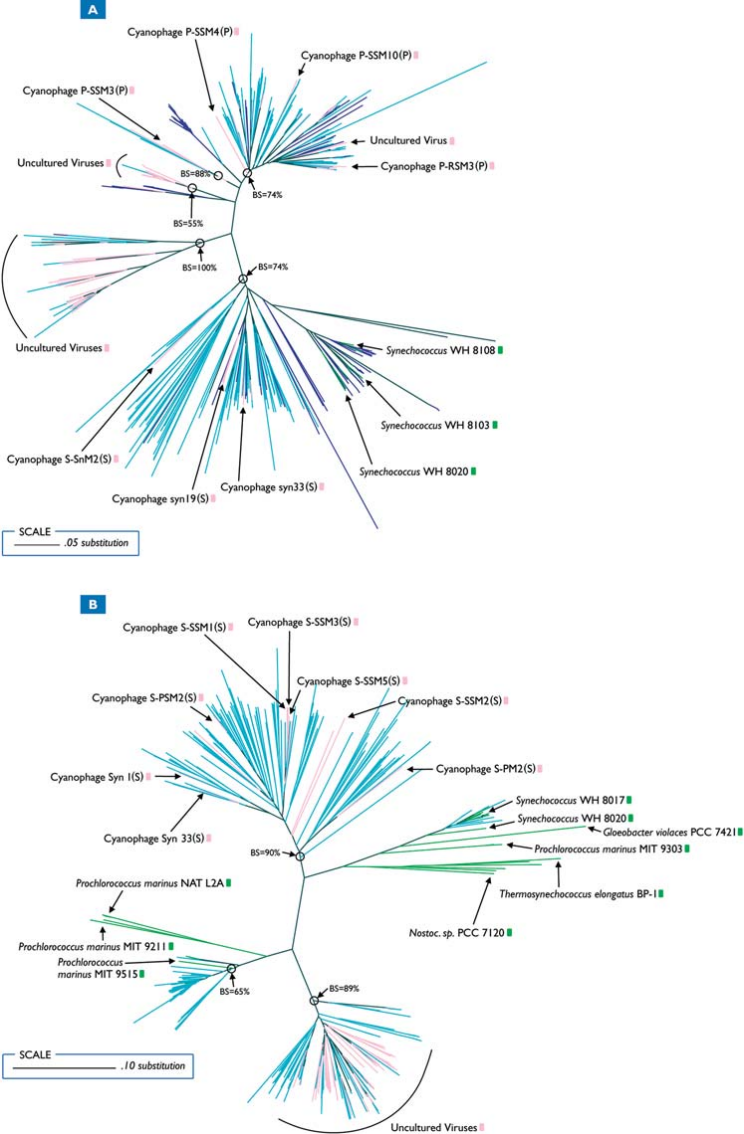
Phylogenetic trees of all GOS and publicly available *psbA* (A) and *psbD* (B) sequences. BS indicates bootstrap values. GOS and public viral sequences are colored aqua and pink respectively. GOS and public prokaryotic sequences are navy blue and lime green respectively.

We performed phylogenetic analyses on all GOS and publicly available nucleotide sequences belonging to the gene families described above in order to test the hypothesis that viral genes have undergone significant evolution since they were first acquired from their hosts. Out of the five PS-related viral gene families, *psb*A and *psbD* exhibited the strongest evidence for highly supported viral clades containing both GOS viral and public viral sequences (Fig. 2A and 2B), further confirming the viral nature of these genes and supporting our hypothesis. GOS *psbA* viral sequences formed multiple coherent clusters containing predominantly *Synechococcus* myoviral sequences, *Prochlorococcus* myoviral sequences, or those from uncultured environmental viruses (Fig. 2A). GOS viral sequences clustered either with *Synechococcus* or *Prochlorococcus* phage sequences, but no clusters contained both. This pattern suggests that the GOS sequences falling within *Synechococcus* or *Prochlorococcus* phage clusters represent novel representatives of phages infective for these groups of cyanobacteria. The clade containing GOS viral and *Synechococcus* viral *psbA* sequences also contained multiple *Synechococcus* cyanobacterial sequences, alluding to the possible origin of viral genes within this cluster. In addition, another highly supported cluster (100% bootstrap support) contained GOS viral sequences and public *psbA* sequences from uncultured marine viruses, but no *Synechococcus* or *Prochlorococcus* viral sequences, suggesting that these viruses are not closely related to known *Synechococcus* or *Prochlorococcus* phages.

**Figure 3:**
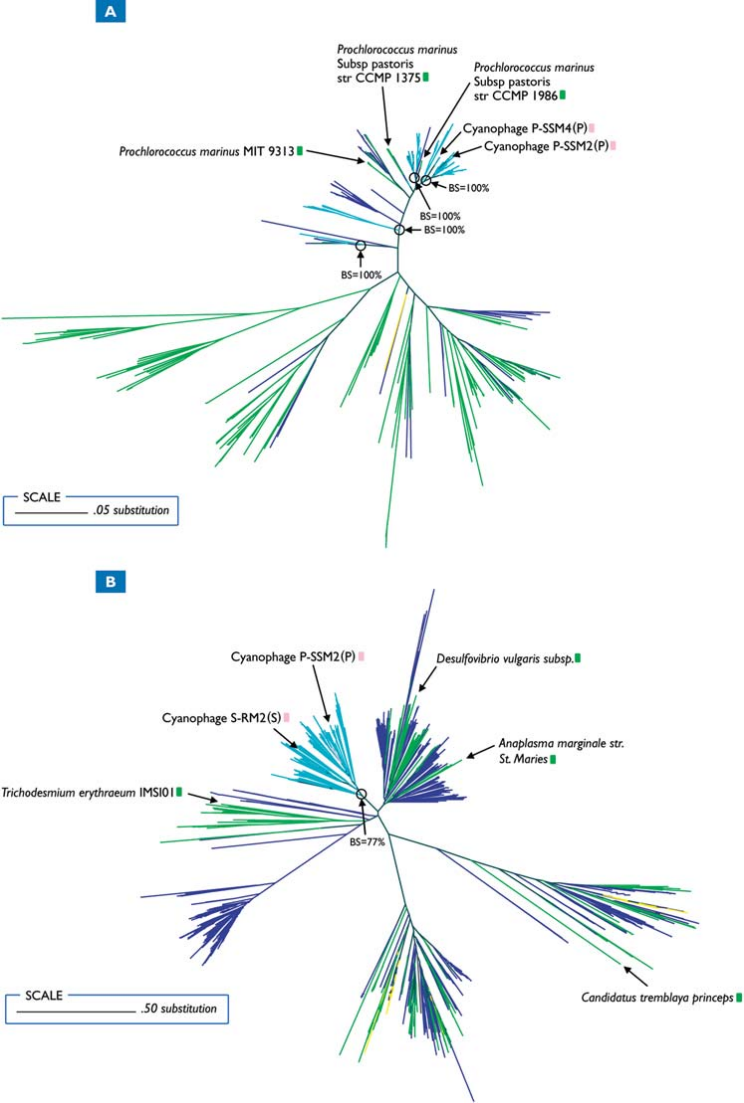
Phylogenetic trees of all GOS and publicly available *pstS* (A) and *talC* (B) sequences. BS indicates bootstrap values. GOS and public viral sequences are colored aqua and pink respectively. GOS and public prokaryotic sequences are navy blue and lime green respectively. GOS eukaryotic sequences are colored yellow.

GOS viral *psbD* sequences also formed coherent clusters with publicly available viral sequences (Fig. 2B). Public viral sequences consisted of either *Synechococcus* myoviruses or uncultured environmental viruses, but no *Prochlorococcus* viruses. The lack of *Prochlorococcus* viral sequences is not unexpected as Sullivan et al. (2006) [30] demonstrated that 85% of *Synechococcus* myoviruses contained *psbD*, while only 33% of *Prochlorococcus* myoviruses contained the gene. Similar to *psbA* sequences, one highly supported viral *psbD* clade (89% bootstrap support) contained GOS and uncultured viral sequences exclusively. GOS viral *talC* and *pstS* sequences also formed well supported clusters with public viral sequences (Figs. 3A and 3B). GOS viral *pstS* sequences (Fig. 3A) formed several small clusters (all supported 100%); one containing *Prochlorococcus* viral *pstS* sequences while GOS viral *talC* sequences (Fig. 3B) formed one major cluster (77% bootstrap support) containing both *Synechococcus* and *Prochlorococcus* viral sequences. Although it's tempting to draw conclusions regarding the specific microbial origin of the viral genes based on their proximity to known bacterial sequences, this would be difficult and likely inaccurate in the absence of specific host-range information [30].

### Distribution of viral genes of host origin across sampling sites

Viral genes of host origin were identified in varying abundances across all sites sampled (Fig. S1, S2, S3, S4, S5, S6, S7, S8), indicating the prevalence and widespread nature of this phenomenon in aquatic ecosystems. Viral PS-related gene families were generally the most abundant at the eastern Pacific sampling locations (GS20-GS30) with the exception of *petE* sequences which were the most abundant at GS51, a coral reef atoll in the South Pacific Gyre. Viral *talC* sequences were also highly abundant in the eastern Pacific and were highest at a fringing reef off of Coco's island, Ecuador (GS25). The distributions of viral phosphorous-related genes families differed with respect to their abundances, with *pstS* sequences peaking at a sampling location northeast of Colon, Panama (GS19) and *phoH* sequences peaking at Punta Cormorant (GS33), a hypersaline lagoon located on Floreana island in the Galapagos. Positive correlations were observed between the distribution of myovirus sequences and all viral gene families of host origin with the exceptions of *pstS* and *petE* sequences (Table S7). Positive relationships were also noted between the distributions of P-SSM4-like sequences and four of eight viral gene families including *psbA*, *hli*, *talC* and *pstS* (Table S7). Since the majority of *Myoviridae* scaffolds in our data are cyanophage-related, our observations suggest that these viral gene families represent signature cyanophage genes as suggested by Sullivan and colleagues (2005) [27]. The fact that *pstS* sequences were not correlated with myovirus distribution, but were positively related to viral sequences that were closely related to the cyanomyovirus P-SSM4 suggests that cyanomyoviruses may be more likely to carry this gene than myoviruses infecting heterotrophic bacteria. Although the distribution of *Prochlorococcus*-related sequences was positively correlated with the *pstS* and *hli* sequences, these relationships were weaker than those observed with P-SSM4-like sequences (Table S7).

We currently know very little about the biotic and abiotic factors that influence the acquisition of host metabolic genes by certain viruses in aquatic environments. The data presented here allowed us to evaluate the impact of various environmental parameters on the occurrence and geographic distribution of viral genes of host origin captured within GOS samples. While all of the viral gene families investigated were positively correlated with water temperature, only certain viral gene families were correlated with other environmental parameters such as salinity, overall water depth and calculated trophic status indices (TSI) [47], [48] (Table S7). Negative relationships were observed between TSI and 5 of 8 viral gene families including *psbA*, *psbD*, *hli*, *speD* and *pstS* (Table S7). Alternatively, positive correlations were noted between salinity and viral *pstS* sequences as well as overall water depth and *pstS*, *hli* and *psbD* sequences (Table S7). Based on the geographic and statistical data, the majority of host-derived viral genes increased in relative abundance from temperate, misotrophic waters to tropical, oligotrophic waters, similar to the trends observed for P-SSM4-like and *Prochlorococcus*-related sequences. The positive relationships between viral *pstS* sequences, salinity and overall water depth were not unexpected as nutrient concentrations (e.g. PO_4_) often decrease with distance from the coast (as water depth increases) due to less input from land-based sources; while salinity increases due to a decrease in fresh-water influence. Although a weak positive correlation was observed between the distributions of viral *pstS* and viral *phoH* sequences (rs = 0.35; P = 0.02); similar trends with regard to salinity and water depth were not observed, suggesting that different environmental pressures may influence the acquisition of these genes by viruses.

### Fragment recruitment of viral sequences

**Figure 4:**
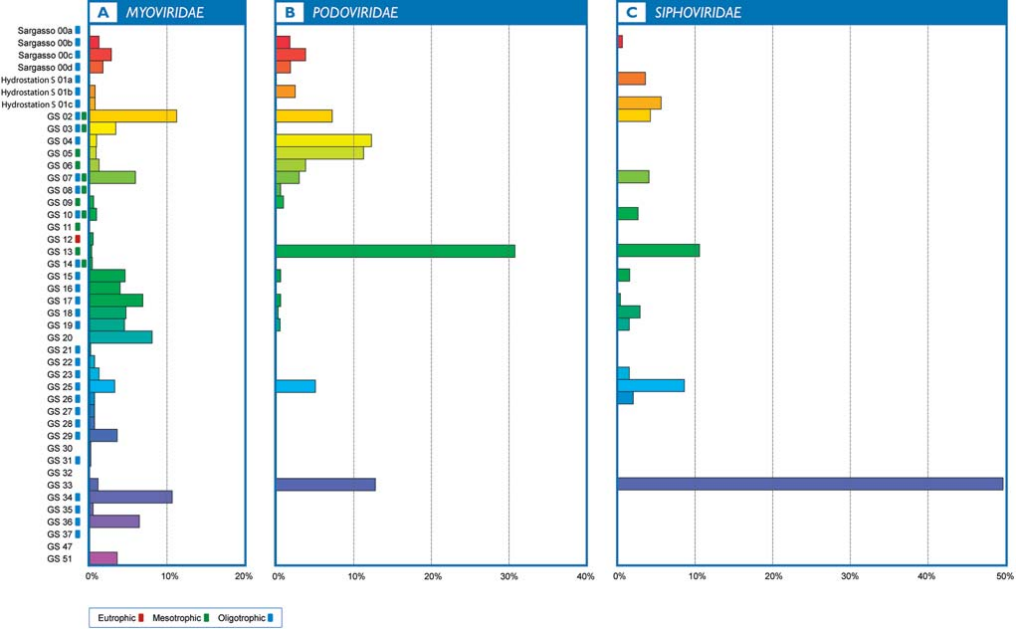
Distribution of GOS myovirus (a), podovirus (b) and siphovirus (c) sequences residing on scaffolds ≥5 kb across sampling locations. The x-axis represents the relative abundance of normalized sequences per site, displayed as a percentage. Sampling locations and trophic status are displayed along the y-axis. Blue boxes indicate oligotrophic conditions, green boxes indicate mesotrophic conditions and red boxes indicate eutrophic conditions. Samples that are in close geographic proximity to each other share similarly colored histogram bars.

When comparing GOS viral sequences to the assembled GOS data, sequences related to the three families of tailed phage, *Myoviridae* (contractile tail), *Podoviridae* (short tail) and *Siphoviridae* (long, non-contractile tail) represented the largest proportion of high identity matches (98% identity to the reference) to known viruses (n = 8,964 sequences) (Table S8), comprising 90%, 6% and 4% of phage sequences residing on scaffolds >5 kb respectively. Viral sequences that were most closely related to *Phycodnaviridae* (algal viruses) were not highly abundant, but did comprise the largest proportion of high identity matches to viruses infecting eukaryotes in our samples (n = 2,696 sequences) (Table S9). As we expected, little recruitment to fully sequenced phycoviral genomes (n = 5) was observed since the great majority of host cells would have been retained on the larger size membrane filters. While the collected samples spanned multiple environmental gradients, statistically significant correlations between the distributions of tailed phage sequences and environmental parameters such as water temperature, salinity, overall water depth and TSI were not observed. *Myoviridae*-related sequences were ubiquitously distributed among sampling sites and were most prevalent at the tropical, oligotrophic Caribbean Sea sampling locations (GS15-GS19) and at Lake Gatun (GS20), located within the Panama Canal (Fig. 4). In contrast, podovirus and siphovirus sequences exhibited more site-specific distributions (Fig. 4). Podovirus sequences were most prevalent in the temperate, primarily mesotrophic waters collected off of Canada and the US eastern seaboard, peaking in abundance at a sample collected in close proximity to Nags Head, N.C. located immediately west of the Gulf Stream (GS13). The largest proportion (85%) of siphovirus sequences originated from the hypersaline lagoon, Punta Cormorant, on Floreana Island in the Galapagos (GS33) and the remaining sequences were recovered from samples collected in temperate, mesotrophic waters and tropical, oligotrophic waters.

**Figure 5:**
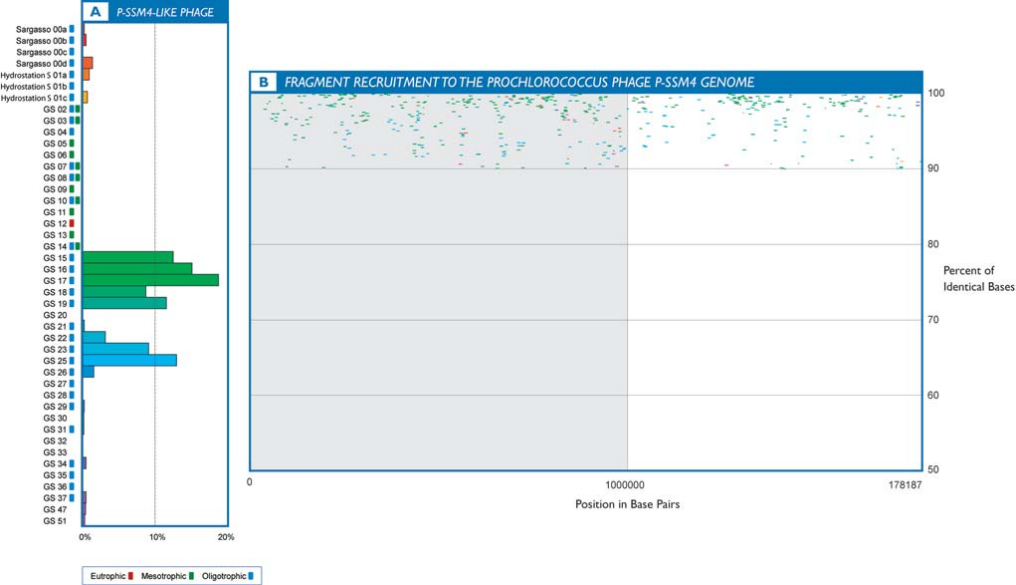
Recruitment of GOS sequences to the *Prochlorococcus* myophage P-SSM4 genome at 90% identity (B) and the distribution of these sequences across sampling locations (A). The x-axis of the recruitment plot shows the position of sequence reads along the complete genome in base pairs and the y-axis represents percent identity. The x-axis of the histogram represents the relative abundance of normalized sequences per site, displayed as a percentage. Sampling locations and trophic status are displayed along the y-axis. Blue boxes indicate oligotrophic conditions, green boxes indicate mesotrophic conditions and red boxes indicate eutrophic conditions. Samples that are in close geographic proximity to each other share similarly colored histogram bars.

When the GOS data was compared to fully sequenced marine viral genomes, only a single phage genome, P-SSM4, displayed substantial abundance of high identity matches. P-SSM4 is a cyanomyovirus that has been experimentally shown to infect two high-light adapted and two low-light adapted strains of *Prochlorococcus* in culture [23], [27]. Our analysis revealed that across sampling sites, the P-SSM4-like phage is represented at approximately 2.8× coverage at a 90% identity cut-off value over the entire length of its genome (Fig. 5). The co-distributions of P-SSM4-like phage sequences with sequences attributed to the dominant ecotype of *Prochlorococcus* in GOS samples, as determined by fragment recruitment to the *Prochlorococcus* MIT9312 strain, [41] were positively correlated (rs = 0.74, P<0.01) (Fig. S9). This suggests that the P-SSM4-like phage may also infect *Prochlorococcus*. Furthermore, myovirus and P-SSM4-like sequences were positively related (rs = 0.559; P = 0.02), suggesting that this phage comprised a proportion of myovirus populations across sampling sites.

According to the BLAST-based taxonomic assignment of phage scaffolds, no *Myoviridae* scaffolds that were >5 kb in length were taxonomically assigned to P-SMM4 despite its prevalence in the GOS data. A large degree of microdiversity between P-SSM4-like phage genomes would be sufficient to prevent their assembly into scaffolds of significant length for analysis (>5 kb) despite high sequence abundance. This is evidenced by the number of high identity matches to the P-SSM4 genome (n = 496; 90% identity) and the abundance of scaffolds that are <5 kb in length assigned to the P-SSM4 phage (n = 775). The GOS dataset consists of paired-end (i.e. mated) sequences derived from individual clones. Upon closer analysis of the sequence reads that had missing mates (i.e. where one paired read aligns to a reference genome while the other does not [41]) associated with the P-SSM4 reference genome, only sixteen reads did not align or were only partially aligned to the reference. This indicated the presence of small deletions or replacements in the P-SSM4-like genomes. When the missing mates were compared (BLASTx) to the NCBI non redundant database, the great majority of top hits were identified as *Prochlorococcus* phage-related proteins or to strains of *Prochlorococcus* itself, suggesting that these clones are prophage-related or that viral sequences were closely related to their host.

The proportion of infected cells at different sampling sites can be projected based on the site-site distribution of P-SSM4-like phage and *Prochlorococcus*-associated sequences. This estimation requires that we assume that most of the P-SSM4-like viral DNA sampled over the course of the expedition was collected from infected *Prochlorococcus* cells rather than from viruses captured through non-specific interactions with the filters. We considered sampling sites where 1) both virus and host- associated sequences were present and 2) the estimated depths of coverage of virus and host genomes were at least 0.1%. The average percentage of this cyanobacterial population that is potentially infected by P-SSM4-like phages is 6.7±4.2% (range = 2.8–13.3%) (Table 2). A similar range of cyanophage infection was determined for samples collected at the Hawaii Ocean Time series ALOHA station [37]. Despite the observed differences in the estimated percentage of infected *Prochlorococcus* cells across sampling sites, all sites were classified as oligotrophic according to their calculated TSIs. This suggests that the nutrient status of the host cells at these locations did not influence the success of lytic infection at the times of sampling [49], [50]. Based on the known host range of the P-SSM4 phage and the co-distribution of high density PSSM4-like phage and *Prochlorococcus*-associated reads in the GOS data, our estimates of cyanophage infection suggest that phages closely related to P-SSM4 may exert a controlling influence on the most abundant ecotype of *Prochlorococcus* in our samples.

**Table 2:** Estimated levels of infection of *Prochlorococcus* by P-SSM4-like phages.

Site	% of Infected Cells	Site Description
15	4.9	Dry Tortugas, Florida
16	13.3	Gulf of Mexico
17	11.9	Yucatan Channel
18	2.8	Rosario bank, Honduras
19	4.9	Northeast of Colón, Panama
23	3.2	30 nm from Cocos Island, Costa Rica
25	6.3	Dirty Rock, Cocos Island, Costa Rica
	**Avg = 6.7±4.2**	

## Discussion

Our investigations of viral sequences identified within the microbial fractions of samples collected as part of the GOS expedition provided a unique opportunity to characterize microbial-associated viral populations over a large geographic transect. Previous metagenomic investigations of aquatic viral communities have primarily focused on the viral-sized fraction of water samples with the exception of Delong et al. (2006) [37] and Venter et al. (2004) [34]–[36], [40]. In contrast, this is the first study of significant magnitude to conduct simultaneous exploration of microbial and viral sequence space, within the same size fraction, collected from a global set of aquatic samples. We have presented strong evidence for the significant occurrence and geographically widespread distribution of environmentally important viral genes of host origin in aquatic ecosystems. Furthermore, examination of viral sequences residing within the microbial fraction of GOS data revealed novel observations regarding distributional patterns of viral families over significant environmental gradients and potential interactions between highly abundant bacteriophage and host organisms.

Although viruses are generally much smaller in size than their hosts, there are a number of reasons why viral sequences can be detected within the microbial fraction of seawater. First, viruses exceeding a particular filter size cutoff (generally 0.1 µm–0.22 µm) in particle size will automatically be retained due to their geometry; and such viruses have been identified in increasing numbers through oceanic and freshwater sampling efforts [51]. However, despite the presence of viruses capable of infecting diverse groups of eukaryotic hosts, the overwhelming majority of viruses in marine ecosystems are bacteriophages which are generally less than 0.2 µm in size [52]–[56]. Many “free-living” phages, or those that are not in physical contact with their host cells, would easily pass through a small pore-size membrane filter. However, phages that are in association with their host cells through either the lytic infection cycle or as prophages are likely to be captured as part of the greater microbial community. Additionally, non-specific interactions between viral particles and the microbes retained on filters may result in the detection of viral DNA on filters, especially as the filters become more loaded with biomass during large volume filtration. Since only a small proportion of GOS viral sequences were prophage-related, it appears that the majority of the viral sequences identified within the microbial fraction of data originated from actively replicating viral particles.

Viral genome sequencing and targeted amplification studies have led to the important discovery that phage encode environmentally relevant genes of host origin [27], [29], [30], [32], [44], [57]. The general consensus that has emerged from these previous investigations is that the acquisition of host genes by viruses involved in metabolic processes such as photosynthesis, as well as carbon and phosphate metabolism may provide fitness advantages to the phage by maintaining critical pathways during the infection and replication processes [27], [30], [31], [57]. Previous studies have focused on the occurrence of specific host-derived viral gene families (primarily *psbA* and *psbD*) within phage isolates and natural viral populations collected from a limited number of geographic locations [30], [33], [57]. Prior to this study, nothing was known regarding the prevalence or distribution of viral genes of metabolic significance throughout the world's oceans. Through our analyses of GOS viral sequences, we have provided compelling evidence that environmentally significant viral genes of host origin are not only widely distributed over a vast array of aquatic ecosystems, but that the viruses carrying these genes can comprise significant proportions of aquatic viral communities.

Our analyses of host-derived viral sequences suggests that viruses likely play a more substantial role in environmentally relevant metabolic processes than previously recognized such as the conversion of light to energy, photoadaptation, phosphate acquisition, and carbon metabolism. Prior to this study, only a very limited number of phage genomes were know to carry the metabolic genes (with the exception of *psbA* and *psbD*) detected in our samples [27]–[29], [57], [58] and limited data existed on their occurrence and distribution in environmental samples [30], [33], [37]. Quantitative PCR analysis of the host-derived viral genes presented here indicated that these they are not only present in the viral fraction of aquatic samples, but also highly abundant. We are uncertain if the viral genes present in our data were actively expressed at the time of sampling. However, experimental evidence does exist for the expression of viral *psbA* and *hli* genes in culture and viral *psbA* genes in the environment, suggesting that host-derived viral genes actively contribute to host cellular processes in some capacity [32], [59]. It is unlikely that we would have observed such a high occurrence and broad geographical distribution of these viral genes if they provided no ecological advantage to the virus, and were simply the result of accidental DNA packaging. Rather, it is hypothesized that viral acquisition, retention, and expression of certain host genes results in an overall increase in fitness by supporting maximal viral replication and potentially expanding host ranges [27], [30], [32].

In many cases, analysis of GOS and publicly available viral sequences indicated that the viral sequences had undergone significant evolution since their time of acquisition. Supporting this hypothesis, Zeidner et al. (2005) [33] concluded that cyanophage *psbA* sequences evolve at an increased evolutionary rate compared to cyanobacterial sequences based on models of nucleotide and codon evolution. Furthermore, an in depth analysis of cultured cyanophage and environmental viral *psbA* and *psbD* sequences suggested that these viral genes have diversified since their time of acquisition and potentially serve as a genetic reservoir for their hosts [30]. With the exception of *psbA*, *psbD* and *hli*, little attention has been directed at the detection and phylogenetic analysis of viral gene families of host origin involved in aspects of photosynthesis or other forms of cellular metabolism [30], [31], [33]. We have observed similar patterns in the phylogenetic distributions of viral *talC* and *pstS* genes as those observed for *psbA* and *psbD* in that viral genes tend to form coherent clusters. As more viral genes of host origin are detected in environmental samples and cultured phage genomes, their evolutionary relationships with host genes should become clearer.

The ubiquitous distribution of myovirus sequences suggests that these tailed phages are capable of sustaining impressive populations by infecting a diverse range of host cells, distributed over a wide geographic area. Cyanomyophages have been experimentally shown to have broader host ranges than cyanopodo or cyanosiphophages [23], [30]. Furthermore, our data indicate that the vast majority of myovirus sequences in the GOS data originated from cyanophages, suggesting that these phages may be partially responsible for the high levels of cyanobacterial diversity observed across GOS samples [41]. Based on the statistically significant relationship between *Prochlorococcus* and the P-SSM4-like phage sequences and estimates of phage infection, our data support the suggestion that P-SSM4-like phages may influence the abundance, distribution and diversity of one of the most dominant components of picophytoplankton in oligotrophic oceans. In contrast, the collective abundance and distributions of GOS podo and siphovirus sequences were significantly lower and more geographically constrained than those attributed to myoviruses, supporting the notion that local geographic conditions influence the composition of viral assemblages in marine ecosystems [36].

Large-scale metagenomic analyses of marine microbial communities have resulted in findings that have substantially increased our understanding of how microbes interact with their environment and the ecological implications of such interactions [37], [39], [41], [42], [60]. From a metagenomic standpoint, little attention has been focused on the co-occurrence of microbial and viral communities. Our coincident analysis of the microbial and viral sequences that were generated from the microbial fractions of environmental samples has provided us with a unique global perspective on the nature of (primarily) marine virus-host interactions and has stimulated intriguing questions with respect to the evolutionary trajectories of viruses and their hosts. Metagenomic examination of the viral fraction of samples collected as part of the GOS expedition will no doubt complement the discoveries made to date and will likely stimulate a whole new set of hypotheses on its own. As we continue to explore marine microbial and viral diversity and gene complement, we may need to redefine how we view ocean biodiversity and function.

## Materials and Methods

### Sample Collection through Assembly of Sequence Data

A detailed description of the sampling sites and collection methods included in this study is discussed in Rusch et al. (2007) [41]. To summarize, approximately 200 L of seawater was collected from each of 37 new sites along a transect from Halifax, Nova Scotia through the South Pacific Gyre. Although the majority of the samples collected were surface seawater, a few unique samples were collected from environments such as a hypersaline lagoon, a shallow hydrothermal seep and a freshwater lake. Water samples were pre-filtered through a 20 µm nytex screen prior to size fractionation by serial filtration through 3.0 µm, 0.8 µm, and 0.1 µm membrane filters (Pall Life Sciences, East Hills, NY). A separate viral fraction was concentrated by tangential flow filtration (TFF) using a Pellicon housing (Millipore, Bedford, MA) fitted with a Biomax-50 (polyethersulfone) cassette filter (50 Kda pore size). Filters were vacuum sealed with 5 ml of sucrose lysis buffer (20 mM EDTA, 400mM NaCl, 0.75 M Sucrose, 50mM Tris-HCl, pH 8.0) and frozen at −20°C on the vessel until shipment back to the Venter Institute, where samples were transferred to a −80°C freezer until DNA extraction. Accompanying physical-chemical data was collected with a YSI Model 6600 multiparameter instrument. Detailed methods describing DNA isolation, library construction, template preparation, automated cycle sequencing and metagenomic assembly can be found in Rusch et al. (2007) [41] and Venter et al. (2004) [40].

Although assembly was conducted with stringent parameters, some rate of misassembly must be foreseen, and any observation based on a single assembly would have to be viewed with some caution. However, results described here are based on patterns observed in multiple assemblies. Moreover, as a consequence of the stringent assembly parameters and the remarkable genetic diversity present in the GOS dataset, more than half of the assemblies that were given a “viral” taxonomic assignment (see below) were in fact trivial assemblies consisting of a single read or a pair of mated reads. Instances of specific protein families described in the results were checked to verify that they were distributed approximately uniformly among such trivial assemblies and true scaffolds composed of data from multiple clones. Thus, potential concerns regarding systematic assembly artifacts can be allayed: most if not all of the results described here could be demonstrated independent of the assembly.

### Taxonomic Assignment of Scaffolds

Scaffolds were given taxonomic assignments according to Yooseph et al. (2007) [39]. Briefly, the top four BLAST matches (E-value<1e-10) of GOS ORFs to NCBI nr were considered, the kingdom of origin for each match was determined and the kingdom votes for each scaffold were subsequently pooled. Each ORF on a scaffold contributed up to four votes. ORFs with fewer than four BLAST matches contributed fewer votes and ORFs with no BLAST matches contributed no votes.

### Fragment Recruitment

A detailed description of the fragment recruitment process can be found in Rusch et al. (2007) [41]. In brief, a fully sequenced genome or scaffolds from the GOS assembly was used to “recruit” GOS sequence reads in order to determine how similar or different they are with respect to one another. Using the assembled GOS data as the reference, the distribution of deeply covered viral sequences contained within 420 scaffolds longer than 5kb were examined. An example of the BLAST-based information that was generated for all viral scaffolds, including scaffold length (all scaffolds had to be ≥5 kb for further examination), the overall taxonomic assignment of the scaffold (viral or bacterial), and the proportion of all bases that could be attributed to viruses is provided in Table S10. All scaffolds that were taxonomically assigned to viruses were subjected to further analysis. In addition, all publicly available, fully sequenced marine phage genomes were used in the recruitment process in order to determine their representation within the GOS dataset.

### Protein Clustering and Site Abundance Estimates

Protein clusters were produced as part of a global protein exploration study and a full description of the clustering process and parameters are detailed in Yooseph et al. (2007) [39]. Briefly, a sequence similarity based clustering of a comprehensive set of known proteins, together with GOS sequences, was used to predict proteins in the GOS data set and to organize sequences into related groups. The clustering utilized similarity over large portions of sequence length, rather than just domains, and length-based thresholds were incorporated to address fragmentary sequences and to minimize grouping of unrelated proteins. The methods to determine site abundance estimates are described in Rusch et al. (2007) [41]. Briefly, the scaffolds containing the genes of interest were identified, and a vector representing the number of sequences contributed by every sample was generated per gene. All vectors were normalized to account for the total number of GOS sequences per sample.

### Phylogenetic Tree Building

Due to the fragmentary nature of a large fraction of the sequences, only sequences that contributed significantly to the multiple sequence alignment were used in tree building. Sequences were aligned using a modified version of CLUSTALW [61]. When Pfams [62] were available, only those sequences that spanned ≥70% of the Pfam length were input to the alignment program. In the absence of Pfams, only those sequences that had ≥60% non-gaps in the alignment were kept. In addition, all columns that contained >10% gaps were removed. The resulting alignment was used to construct a distance matrix using the protdist program in PHYLIP [63]. A phylogeny was inferred from this distance matrix using a modified version of neighbor-joining that did not allow negative branch lengths (http://www.t10.lanl.gov/billb/related_links.html).

### Neighbor Functional Linkage Analysis

BLAST-based kingdom assignments were inferred for all ORFs occurring on the same scaffolds as the viral proteins of host origin. For the eight viral gene families discussed, all ORFs that occurred on the same scaffolds as the viral genes of host origin were collected and a taxonomic assignment of viral, bacterial, eukaryotic, or archaeal was given to each of the same-scaffold ORFs by a majority vote based on the top four BLAST hits to the NCBI nr database [39]. We took a sampling approach to assess the significance of the occurrence of same-scaffold viral proteins. Protein clusters containing both GOS and publicly available sequences [39] were selected at random, a size-matched sample of ORFs was drawn from the cluster, and the taxonomic identities of the same-scaffold ORFs were inferred. This process was repeated 1,000 times for each of the viral gene families discussed and a P value was computed by determining the proportion of the 1,000 samples that had viral same-scaffold ORFs at least as often as the host-derived viral families. P values less than 0.05 were deemed statistically significant.

### Viral Particle Purification and DNA Extraction

Viral concentrates from GS19, GS20, GS26, GS34 and GS51 were further concentrated prior to cesium chloride purification using Centricon Plus-70 centrifugal filter units (Millipore, MA) according to manufacturer's instructions. Cesium chloride purification of viral particles was conducted according to Sambrook et al. (2001) [64]. Purified viral suspensions were de-salted using Slide-A-Lyzer dialysis cassettes (Pierce, IL) according to manufacturer's instructions. Viral capsids were compromised by treatment with proteinase K (50 µg ml^−1^ final concentration) and SDS (10%w/v) and viral DNA was phenol/chloroform extracted and ethanol precipitated. DNA was hydrated in 1XTE and stored at 4°C until qPCR experiments were performed.

### Primer Design and Quantitative PCR

The viral *psbD*, *petE*, *speD*, *pstS*, *phoH*, and *talC* nucleotide sequences that were recovered from the microbial fraction of GOS data were aligned by gene family using a modified version of CLUSTALW [61]. Sub-groups within each gene family were identified through phylogenetic analysis (neighbor-joining) and manual inspection of the alignments. Consensus sequences, including degenerate positions, were calculated for each sub-group using a PERL script designed for this purpose. Briefly, if the most common nucleotide at an aligned position was prevalent 60% of the time, then it was reported in the consensus. Alternatively, a degenerate code would be reported within the consensus based on the most common two, three, etc…nucleotides at an aligned position. Conserved gap positions within consensus sequences were removed.

Quantitative PCR (qPCR) primers (Table S8) were designed from the consensus sequences using Primer Express software (Applied Biosystems, CA). Software parameters, which normally select for the smallest acceptable PCR amplicon in order to maximize PCR efficiency, were also tailored to minimize the number of ambiguous bases. The majority of primers had no more than one ambiguous base. For each qPCR experiment, an oligonucleotide was synthesized that encoded the entire amplicon or the amplicon minus the sequence between the 3′ ends of the two primers for use as standards and positive controls. qPCR experiments were performed using SYBR Green PCR Master Mix (Applied Biosystems), a primer concentration of 500 nM and 10^−2^ and 10^−3^ dilutions of template DNA (2.5–5.0 ng). Reactions were run on an ABI Prism 7700 Sequence Detection System (Applied Biosystems). PCR amplification conditions included an initial 10 cycles of 15 seconds at 95°C, 30 seconds at 54°C and 70 seconds at 72°C, followed by an additional 30 cycles of 15 seconds at 95°C and 60 seconds at 60°C. Positive amplification results were only reported if copy number within the template DNA exceeded copy number within the no template (negative) control by a factor of 10 or more. Additionally, positive results were only reported if copy number in the 10^−2^ dilution was 3–30 times greater than the 10^−3^ dilution. Due to the use of absolute quantification standards, the data are reported as the number of copies per liter of water. Calculations for these values were made using the Sequence Detection System Software.

### Data Release

The 154,662 viral peptide sequences and viral scaffolds discussed in this paper are available for download via the Cyberinfrastructure for Advanced Marine Microbial Ecology Research and Analysis (CAMERA) website (http://web.camera.calit2.net/cameraweb/detailPage.htmaccCAM_PUB_Williamson08a). In addition, the entire set of GOS scaffolds and annotations are available through NCBI (http://www.ncbi.nlm.nig.gov/entrez/query.fcgidbgenomeprjcmdRetrievedoptOverviewlist_uids13694), and the reads are available through the Trace Archive (http://www.ncbi.nlm.nih.gov/Traces/trace.cgi).

## Supporting Information

Figure S1 Distribution of clustered viral psbA sequences detected in the microbial fraction of GOS data across sampling sites. The x-axis represents the relative abundance of sequences per site as a percentage and the secondary y-axis shows the abundance of sequences, normalized to the total number of reads per site. Sampling locations and trophic status are displayed along the primary y-axis. Blue boxes indicate oligotrophic conditions, green boxes indicate mesotrophic conditions and red boxes indicate eutrophic conditions. Samples that are in close geographical proximity to each other share similarly colored histogram bars.
(9.79 MB TIF)

Figure S2 Distribution of clustered viral psbD sequences detected in the microbial fraction of GOS data across sampling sites. The x-axis represents the relative abundance of sequences per site as a percentage and the secondary y-axis shows the abundance of sequences, normalized to the total number of reads per site. Sampling locations and trophic status are displayed along the primary y-axis. Blue boxes indicate oligotrophic conditions, green boxes indicate mesotrophic conditions and red boxes indicate eutrophic conditions. Samples that are in close geographical proximity to each other share similarly colored histogram bars.
(9.65 MB TIF)

Figure S3 Distribution of clustered viral petE sequences detected in the microbial fraction of GOS data across sampling sites. The x-axis represents the relative abundance of sequences per site as a percentage and the secondary y-axis shows the abundance of sequences, normalized to the total number of reads per site. Sampling locations and trophic status are displayed along the primary y-axis. Blue boxes indicate oligotrophic conditions, green boxes indicate mesotrophic conditions and red boxes indicate eutrophic conditions. Samples that are in close geographical proximity to each other share similarly colored histogram bars.
(9.68 MB TIF)

Figure S4 Distribution of clustered viral speD sequences detected in the microbial fraction of GOS data across sampling sites. The x-axis represents the relative abundance of sequences per site as a percentage and the secondary y-axis shows the abundance of sequences, normalized to the total number of reads per site. Sampling locations and trophic status are displayed along the primary y-axis. Blue boxes indicate oligotrophic conditions, green boxes indicate mesotrophic conditions and red boxes indicate eutrophic conditions. Samples that are in close geographical proximity to each other share similarly colored histogram bars.
(9.66 MB TIF)

Figure S5 Distribution of clustered viral hli sequences detected in the microbial fraction of GOS data across sampling sites. The x-axis represents the relative abundance of sequences per site as a percentage and the secondary y-axis shows the abundance of sequences, normalized to the total number of reads per site. Sampling locations and trophic status are displayed along the primary y-axis. Blue boxes indicate oligotrophic conditions, green boxes indicate mesotrophic conditions and red boxes indicate eutrophic conditions. Samples that are in close geographical proximity to each other share similarly colored histogram bars.
(9.90 MB TIF)

Figure S6 Distribution of clustered viral phoH sequences detected in the microbial fraction of GOS data across sampling sites. The x-axis represents the relative abundance of sequences per site as a percentage and the secondary y-axis shows the abundance of sequences, normalized to the total number of reads per site. Sampling locations and trophic status are displayed along the primary y-axis. Blue boxes indicate oligotrophic conditions, green boxes indicate mesotrophic conditions and red boxes indicate eutrophic conditions. Samples that are in close geographical proximity to each other share similarly colored histogram bars.
(9.77 MB TIF)

Figure S7 Distribution of clustered viral pstS sequences detected in the microbial fraction of GOS data across sampling sites. The x-axis represents the relative abundance of sequences per site as a percentage and the secondary y-axis shows the abundance of sequences, normalized to the total number of reads per site. Sampling locations and trophic status are displayed along the primary y-axis. Blue boxes indicate oligotrophic conditions, green boxes indicate mesotrophic conditions and red boxes indicate eutrophic conditions. Samples that are in close geographical proximity to each other share similarly colored histogram bars.
(9.46 MB TIF)

Figure S8 Distribution of clustered viral talC sequences detected in the microbial fraction of GOS data across sampling sites. The x-axis represents the relative abundance of sequences per site as a percentage and the secondary y-axis shows the abundance of sequences, normalized to the total number of reads per site. Sampling locations and trophic status are displayed along the primary y-axis. Blue boxes indicate oligotrophic conditions, green boxes indicate mesotrophic conditions and red boxes indicate eutrophic conditions. Samples that are in close geographical proximity to each other share similarly colored histogram bars.
(9.63 MB TIF)

Figure S9 Distributions of sequencing reads across sampling locations associated with the dominant ecotype of *Prochlorococcus* in GOS samples (90% identity) (A) and sequencing reads associated with the *Prochlorococcus* myovirus P-SSM4 (90% identity) (B). Table S1 contains descriptions of the sampling stations that correspond with the station identification numbers on the y-axis.
(4.58 MB TIF)

Table S1 (0.03 MB XLS)

Table S2 (0.14 MB DOC)

Table S3 (0.06 MB DOC)

Table S4 (0.04 MB DOC)

Table S5 (0.04 MB DOC)

Table S6 (0.09 MB DOC)

Table S7 (0.03 MB XLS)

Table S8 (0.09 MB DOC)

Table S9 (0.05 MB DOC)

Table S10 (0.04 MB DOC)
