# Supplementary figures and images for: The Sorcerer II Global Ocean Sampling Expedition: Metagenomic Characterization of Viruses within Aquatic Microbial Samples
Source: PLoS One. 2008 Jan 23;3(1):e1456. doi: 10.1371/journal.pone.0001456 (PMC2186209; doi:10.1371/journal.pone.0001456)

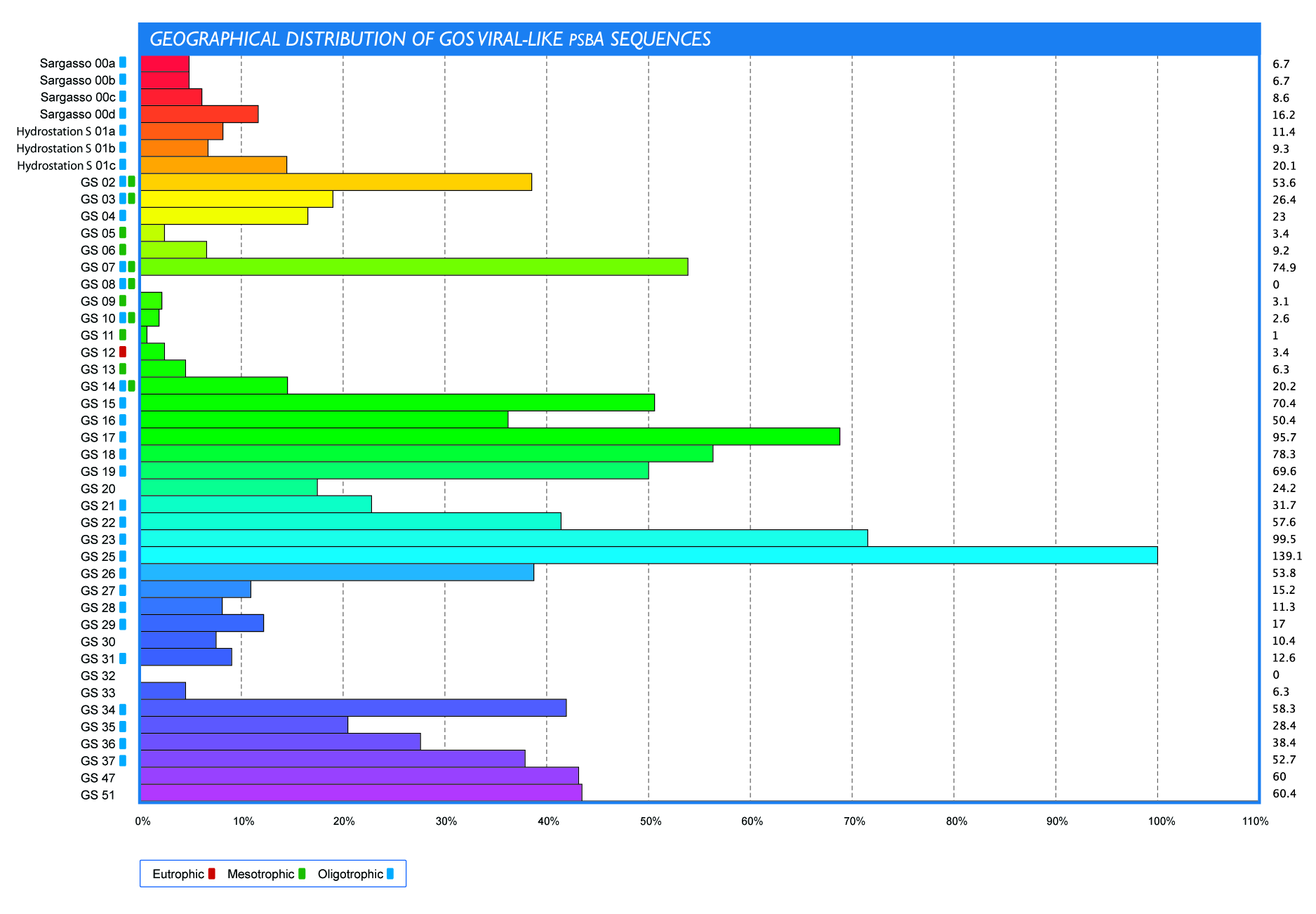

Supplement: Figure S1 — Distribution of clustered viral psbA sequences detected in the microbial fraction of GOS data across sampling sites. The x-axis represents the relative abundance of sequences per site as a percentage and the secondary y-axis shows the abundance of sequences, normalized to the total number of reads per site. Sampling locations and trophic status are displayed along the primary y-axis. Blue boxes indicate oligotrophic conditions, green boxes indicate mesotrophic conditions and red boxes indicate eutrophic conditions. Samples that are in close geographical proximity to each other share similarly colored histogram bars. (9.79 MB TIF) [file pone.0001456.s001.tif]

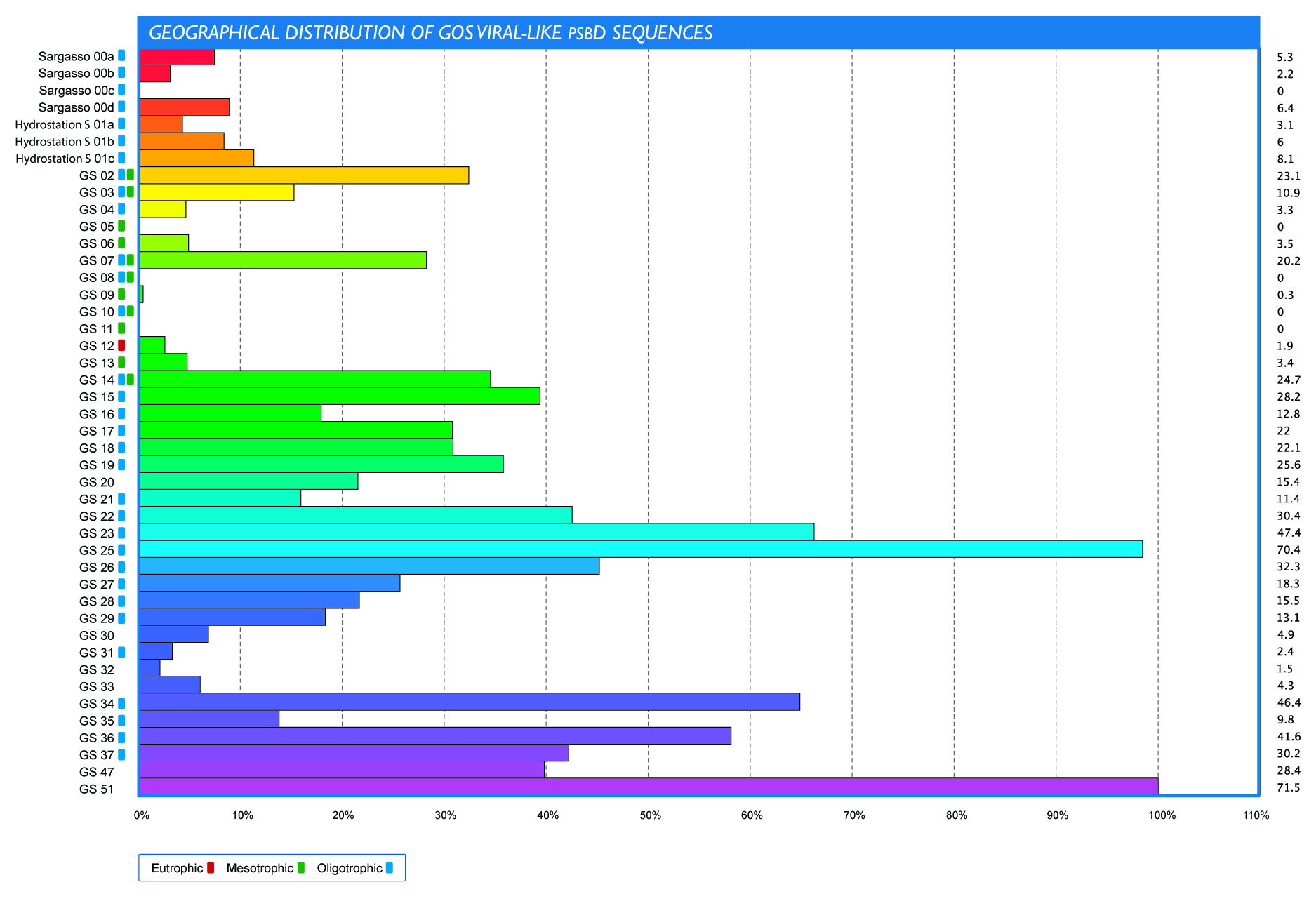

Supplement: Figure S2 — Distribution of clustered viral psbD sequences detected in the microbial fraction of GOS data across sampling sites. The x-axis represents the relative abundance of sequences per site as a percentage and the secondary y-axis shows the abundance of sequences, normalized to the total number of reads per site. Sampling locations and trophic status are displayed along the primary y-axis. Blue boxes indicate oligotrophic conditions, green boxes indicate mesotrophic conditions and red boxes indicate eutrophic conditions. Samples that are in close geographical proximity to each other share similarly colored histogram bars. (9.65 MB TIF) [file pone.0001456.s002.tif]

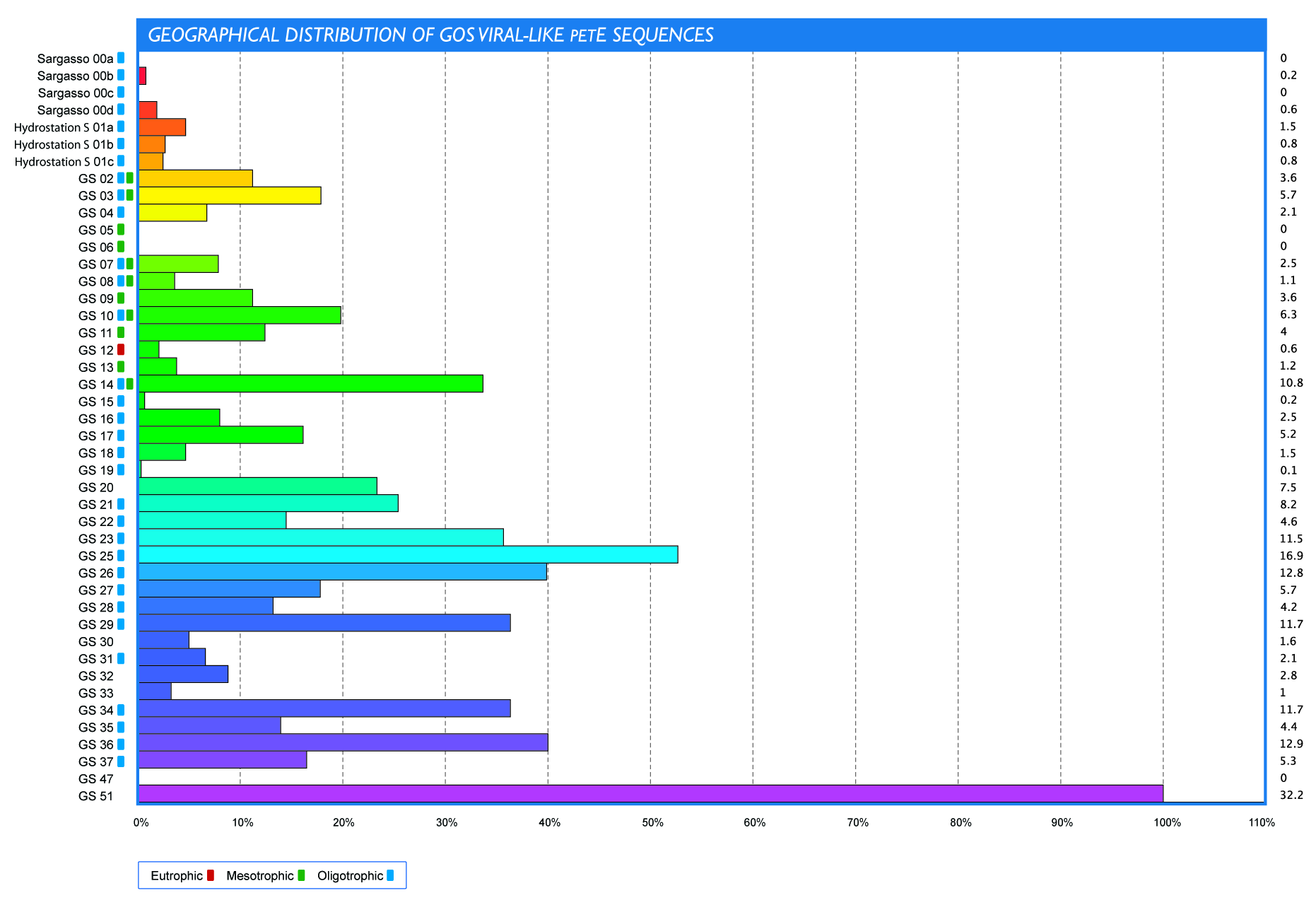

Supplement: Figure S3 — Distribution of clustered viral petE sequences detected in the microbial fraction of GOS data across sampling sites. The x-axis represents the relative abundance of sequences per site as a percentage and the secondary y-axis shows the abundance of sequences, normalized to the total number of reads per site. Sampling locations and trophic status are displayed along the primary y-axis. Blue boxes indicate oligotrophic conditions, green boxes indicate mesotrophic conditions and red boxes indicate eutrophic conditions. Samples that are in close geographical proximity to each other share similarly colored histogram bars. (9.68 MB TIF) [file pone.0001456.s003.tif]

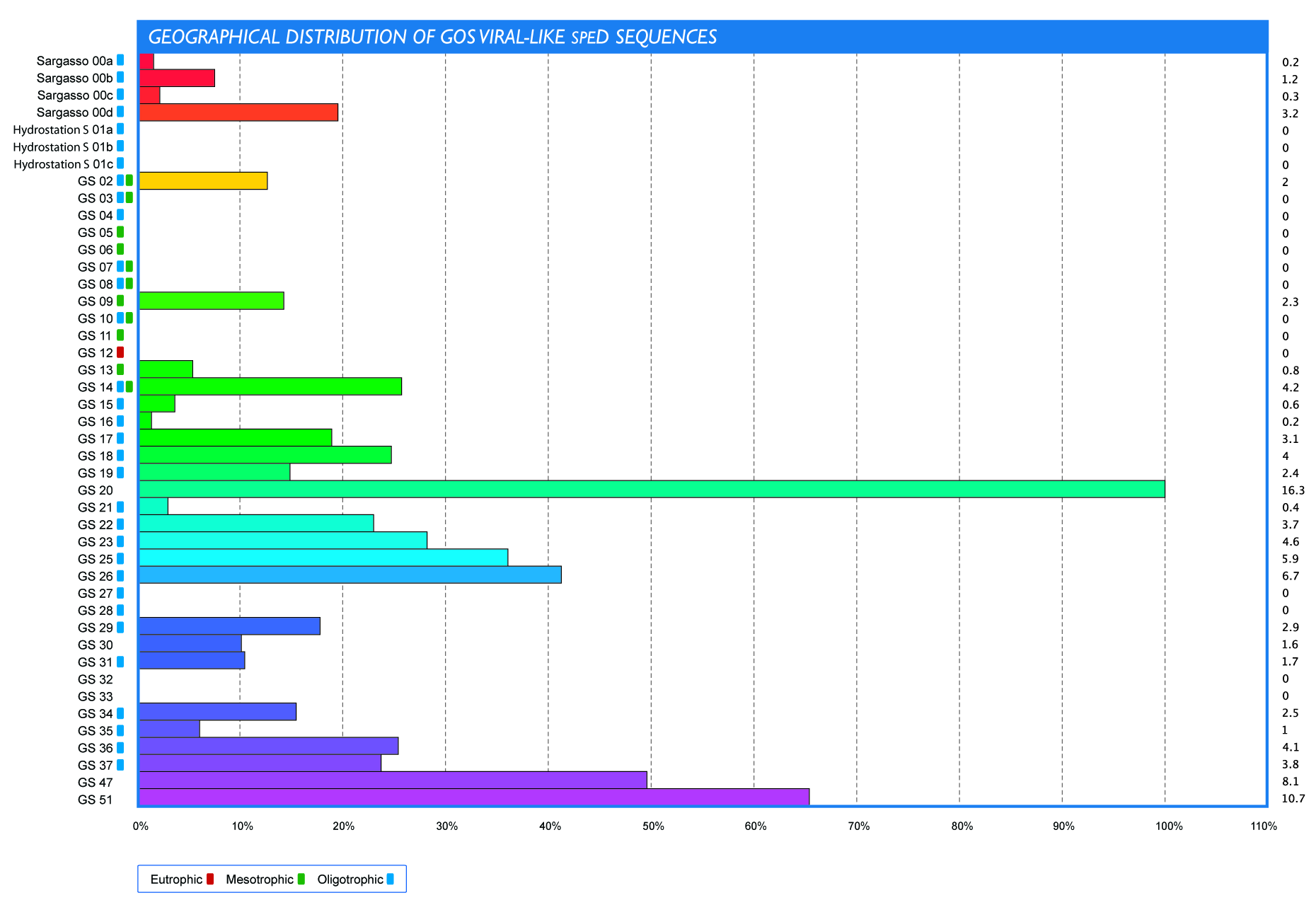

Supplement: Figure S4 — Distribution of clustered viral speD sequences detected in the microbial fraction of GOS data across sampling sites. The x-axis represents the relative abundance of sequences per site as a percentage and the secondary y-axis shows the abundance of sequences, normalized to the total number of reads per site. Sampling locations and trophic status are displayed along the primary y-axis. Blue boxes indicate oligotrophic conditions, green boxes indicate mesotrophic conditions and red boxes indicate eutrophic conditions. Samples that are in close geographical proximity to each other share similarly colored histogram bars. (9.66 MB TIF) [file pone.0001456.s004.tif]

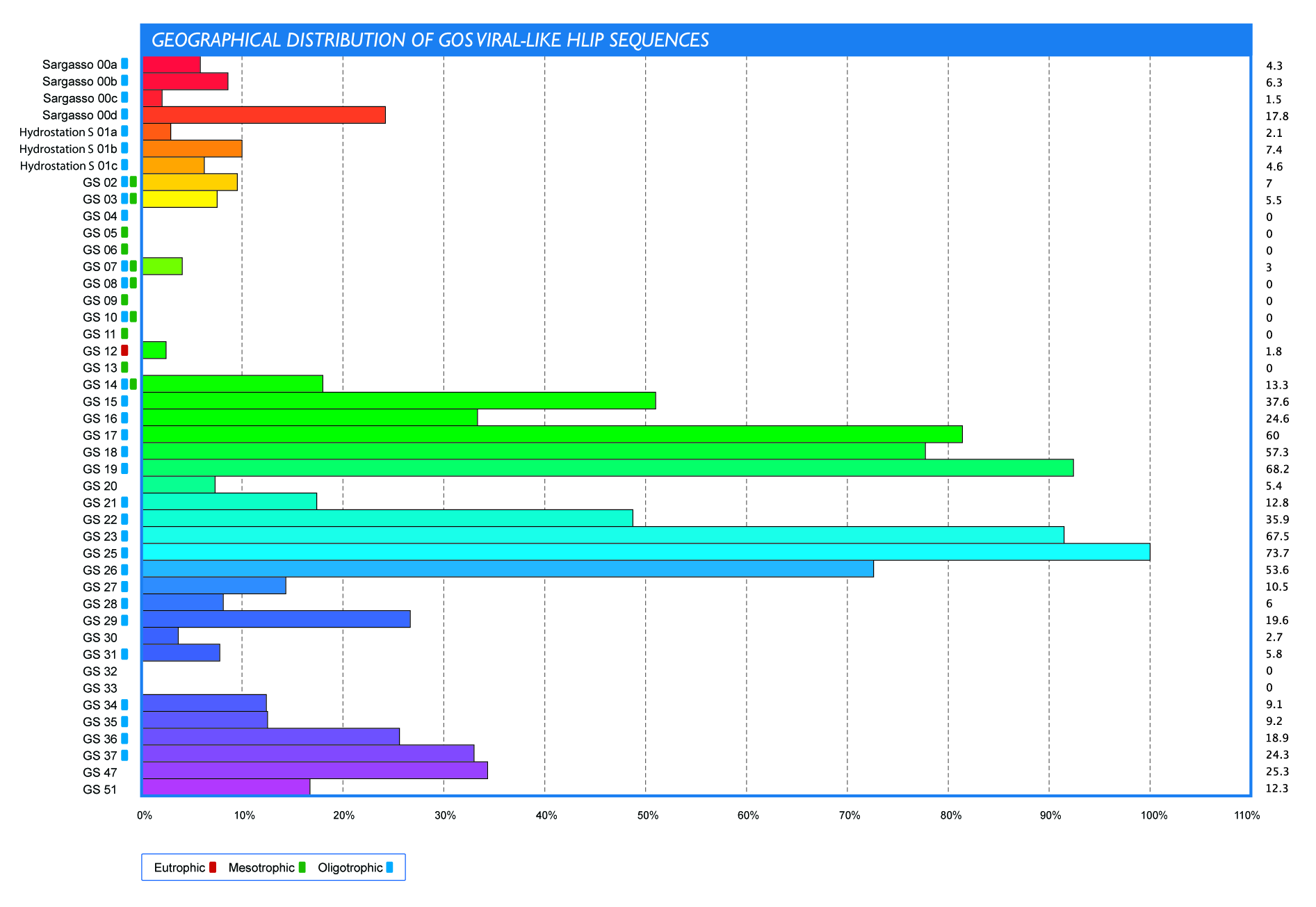

Supplement: Figure S5 — Distribution of clustered viral hli sequences detected in the microbial fraction of GOS data across sampling sites. The x-axis represents the relative abundance of sequences per site as a percentage and the secondary y-axis shows the abundance of sequences, normalized to the total number of reads per site. Sampling locations and trophic status are displayed along the primary y-axis. Blue boxes indicate oligotrophic conditions, green boxes indicate mesotrophic conditions and red boxes indicate eutrophic conditions. Samples that are in close geographical proximity to each other share similarly colored histogram bars. (9.90 MB TIF) [file pone.0001456.s005.tif]

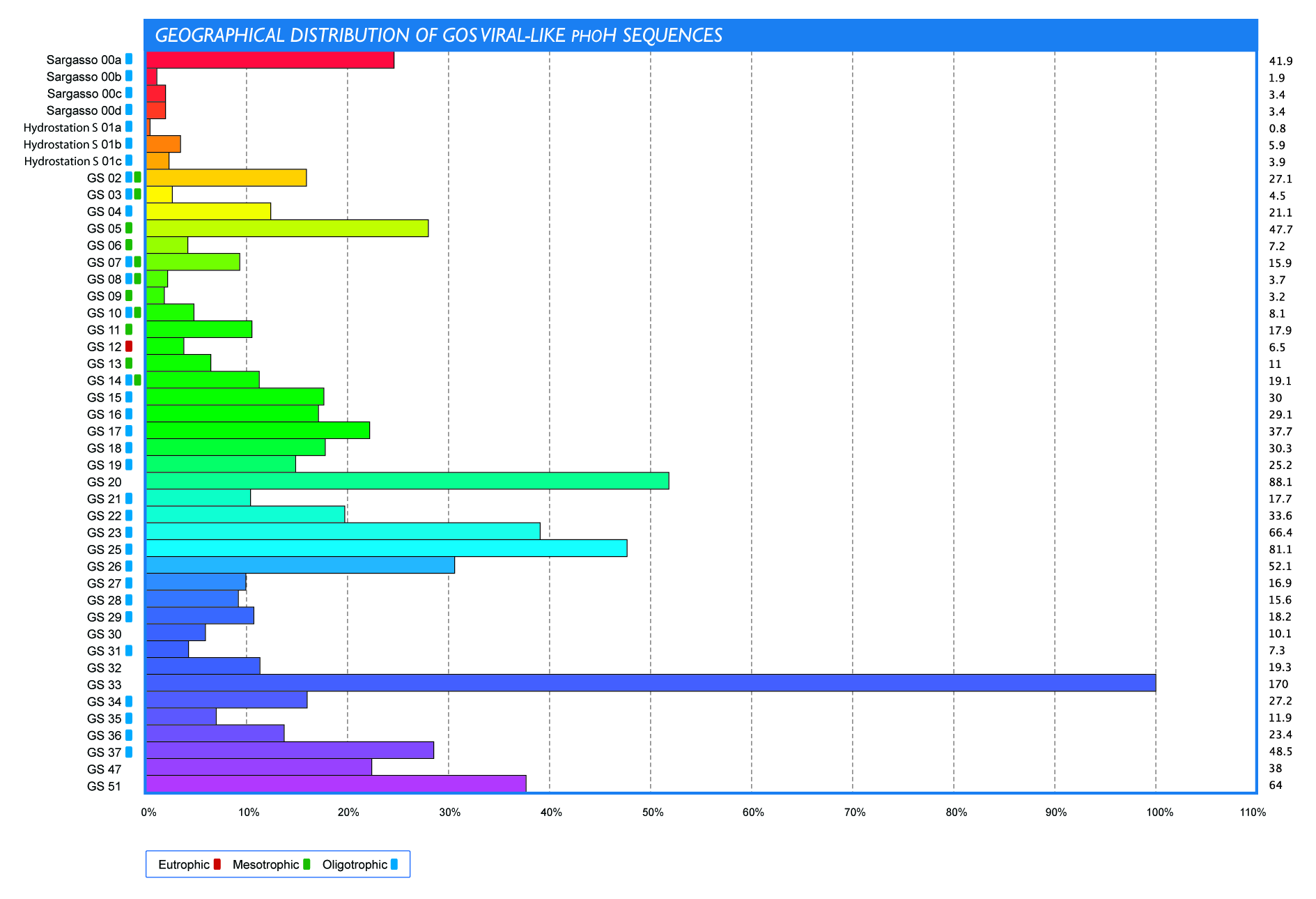

Supplement: Figure S6 — Distribution of clustered viral phoH sequences detected in the microbial fraction of GOS data across sampling sites. The x-axis represents the relative abundance of sequences per site as a percentage and the secondary y-axis shows the abundance of sequences, normalized to the total number of reads per site. Sampling locations and trophic status are displayed along the primary y-axis. Blue boxes indicate oligotrophic conditions, green boxes indicate mesotrophic conditions and red boxes indicate eutrophic conditions. Samples that are in close geographical proximity to each other share similarly colored histogram bars. (9.77 MB TIF) [file pone.0001456.s006.tif]

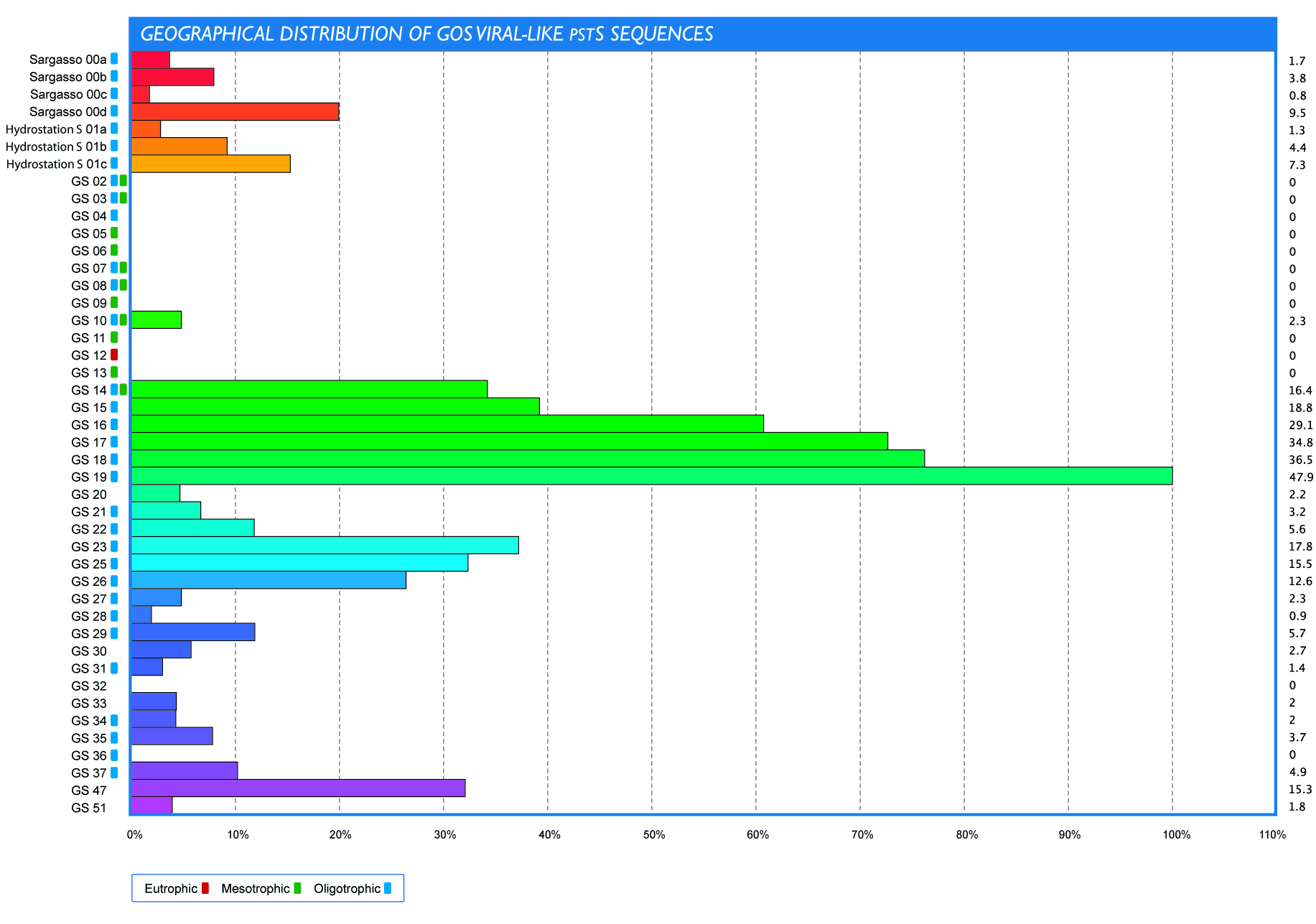

Supplement: Figure S7 — Distribution of clustered viral pstS sequences detected in the microbial fraction of GOS data across sampling sites. The x-axis represents the relative abundance of sequences per site as a percentage and the secondary y-axis shows the abundance of sequences, normalized to the total number of reads per site. Sampling locations and trophic status are displayed along the primary y-axis. Blue boxes indicate oligotrophic conditions, green boxes indicate mesotrophic conditions and red boxes indicate eutrophic conditions. Samples that are in close geographical proximity to each other share similarly colored histogram bars. (9.46 MB TIF) [file pone.0001456.s007.tif]

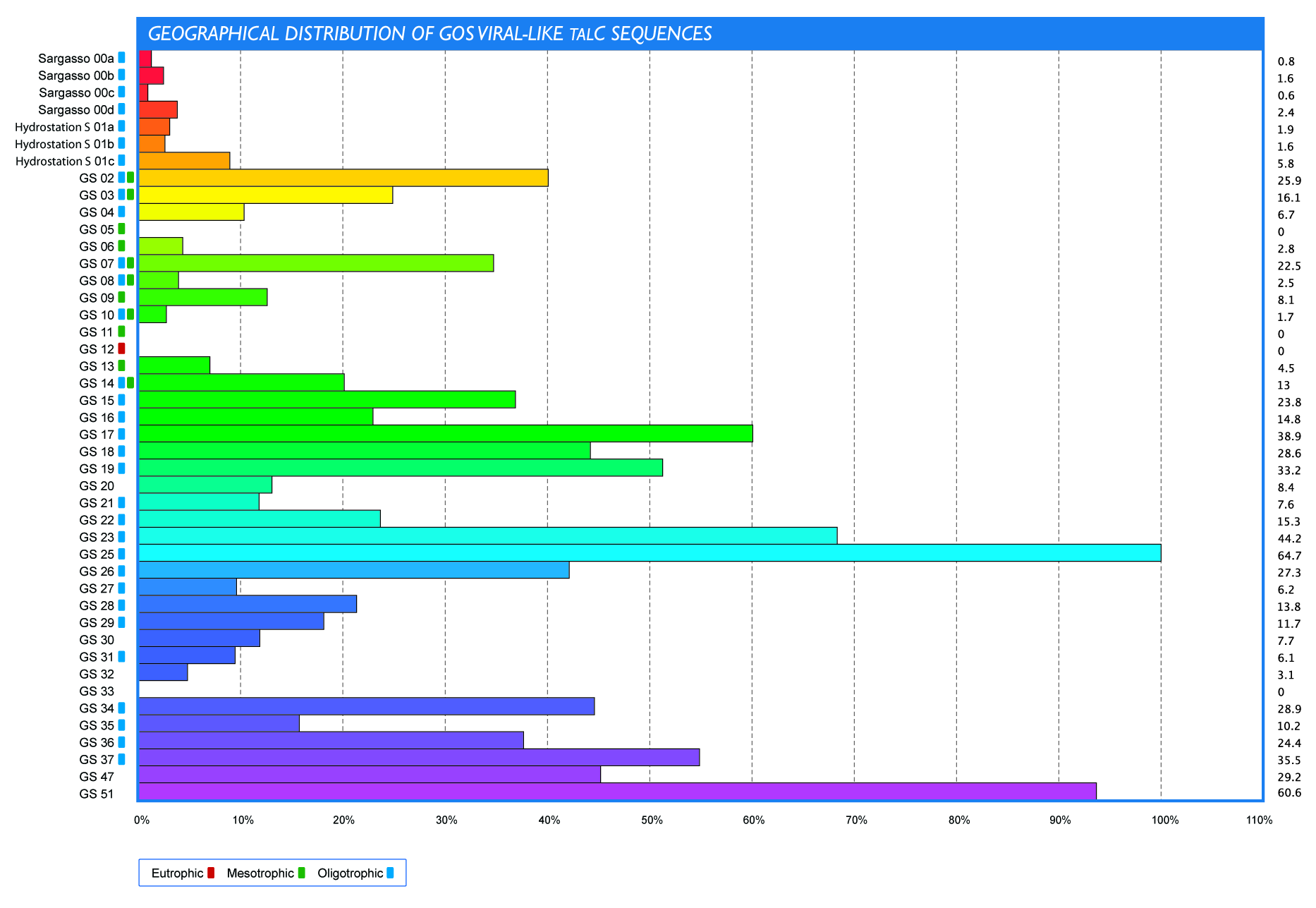

Supplement: Figure S8 — Distribution of clustered viral talC sequences detected in the microbial fraction of GOS data across sampling sites. The x-axis represents the relative abundance of sequences per site as a percentage and the secondary y-axis shows the abundance of sequences, normalized to the total number of reads per site. Sampling locations and trophic status are displayed along the primary y-axis. Blue boxes indicate oligotrophic conditions, green boxes indicate mesotrophic conditions and red boxes indicate eutrophic conditions. Samples that are in close geographical proximity to each other share similarly colored histogram bars. (9.63 MB TIF) [file pone.0001456.s008.tif]

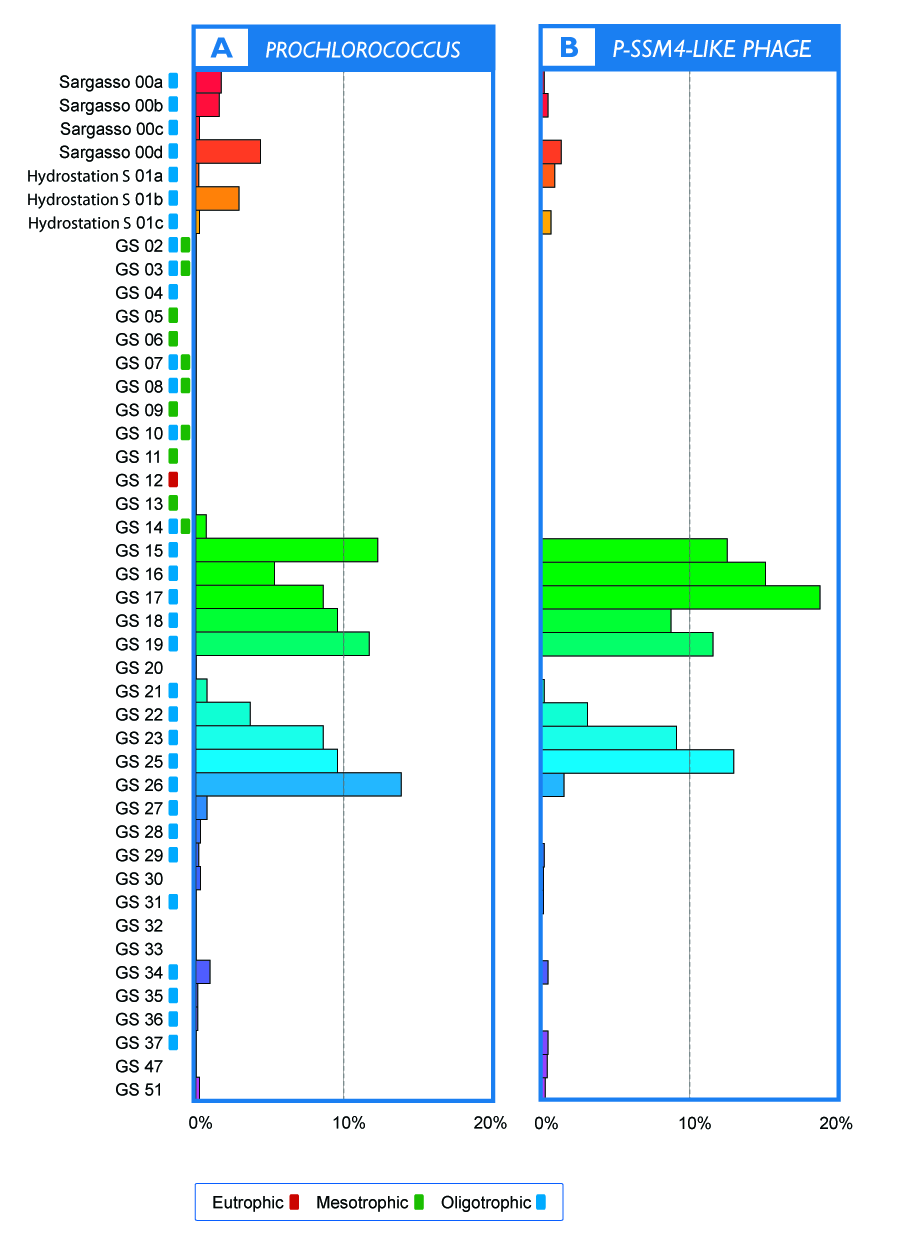

Supplement: Figure S9 — Distributions of sequencing reads across sampling locations associated with the dominant ecotype of Prochlorococcus in GOS samples (90% identity) (A) and sequencing reads associated with the Prochlorococcus myovirus P-SSM4 (90% identity) (B). Table S1 contains descriptions of the sampling stations that correspond with the station identification numbers on the y-axis. (4.58 MB TIF) [file pone.0001456.s009.tif]
