# Supplementary material for: The Sorcerer II Global Ocean Sampling Expedition: Metagenomic Characterization of Viruses within Aquatic Microbial Samples
Source: PLoS One. 2008 Jan 23;3(1):e1456. doi: 10.1371/journal.pone.0001456 (PMC2186209; doi:10.1371/journal.pone.0001456)
Supplement: Table S2 — (0.14 MB DOC) [file pone.0001456.s011.doc]

| Table S2. Viral sequences belonging to GOS phage category. | | | |
| --- | --- | --- | --- |
| Functional Category | # of Clusters | # Sequences | Protein Description (Putative) |
| Phage Infection |  |  |  |
|  | 1 | 472 | LPS biosynthesis - Gycosyltransferase family 25 |
|  | 3 | 595 | Lysozyme/Endolysin |
|  | 1 | 165 | Phage infection protein |
| DNA Replication |  |  |  |
|  | 2 | 802 | ssDNA binding protein |
|  | 1 | 56 | DNA gyrase/topoisomerase |
|  | 2 | 1672 | Phage primase/helicase |
|  | 1 | 32 | Superfamily II DNA/RNA helicase |
|  | 3 | 1779 | DNA replicative helicase |
|  | 5 | 1391 | DNA polymerase |
|  | 1 | 92 | DNA binding protein |
|  | 1 | 289 | DNA clamp loader |
|  | 1 | 739 | DNA sliding clamp |
|  | 1 | 356 | DNA end protector protein |
|  | 4 | 2686 | Exonuclease |
|  | 1 | 471 | Endonuclease |
| DNA Recombination |  |  |  |
|  | 2 | 378 | Site-specific recombinase; Resolvase family |
|  | 1 | 90 | Essential recombination protein |
|  | 1 | 697 | Crossover junction endodeoxyribonuclease |
|  | 3 | 2127 | Recombination endonuclease |
|  |  |  |  |
| DNA Repair | 1 | 111 | dCMP deaminase |
|  | 2 | 321 | NAD-dependent DNA ligase subunit |
|  | 1 | 662 | UVsY recomb, repair and ssDNA binding protein |
|  | 1 | 1137 | DNA recombination/repair endonuclease |
|  | 1 | 1185 | RecA-like protein |
| DNA Modification |  |  |  |
|  | 1 | 96 | Adenine methylase |
| Nucleotide Metabolism |  |  |  |
|  | 1 | 338 | NRD - ribonucleotide reductase partial domain |
|  | 1 | 78 | Deoxynucleotide monophosphate kinase |
|  | 1 | 61 | Dihydrofolate reductase |
|  | 2 | 186 | GTP cyclohydrolase I family protein |
|  | 1 | 252 | Glutaredoxin |
|  | 1 | 727 | MazG nucleotide pyrophosphohydrolase domain |
| Transcription |  |  |  |
|  | 1 | 51 | RNA polymerase |
|  | 1 | 634 | Sigma factor for late transcription |
|  | 1 | 359 | Late promoter transcription factor |
|  | 1 | 35 | Transcriptional regulator |
| Translation |  |  |  |
|  | 1 | 721 | RegA translational repressor |
| Lysogeny |  |  |  |
|  | 2 | 162 | Site-specific recombinase; Phage integrase family |
| Structural Proteins |  |  |  |
| Head |  |  |  |
|  | 2 | 703 | Head-tail connector protein |
|  | 2 | 2468 | Prohead core protein |
|  | 1 | 1055 | Head completion protein |
|  | 1 | 46 | Head decoration protein |
| Neck |  |  |  |
|  | 2 | 2430 | Neck protein |
| Tail |  |  |  |
|  | 2 | 2366 | Tail sheath protein |
|  | 2 | 1822 | Tail sheath stabilizer and completion protein |
|  | 2 | 1176 | Tail tube protein |
|  | 1 | 60 | Tail assembly protein |
|  | 4 | 203 | Tail fiber protein |
|  | 3 | 150 | Tail protein |
|  | 1 | 21 | Pore-forming tail tip protein |
| Baseplate |  |  |  |
|  | 2 | 2760 | Baseplate wedge subunit |
|  | 2 | 1671 | Baseplate wedge |
|  | 1 | 519 | Baseplate hub assembly catalyst |
|  | 2 | 1791 | Baseplate hub subunit |
| Capsid |  |  |  |
|  | 2 | 518 | Minor capsid protein |
|  | 1 | 2172 | Major capsid protein |
|  | 1 | 233 | Capsid protein |
|  | 2 | 2610 | Capsid assembly protein |
| Other Structural |  |  |  |
|  | 1 | 21 | Protein of morphogenic function |
| DNA Packaging |  |  |  |
|  | 8 | 6006 | Terminase subunit |
| Cell Lysis |  |  |  |
|  | 1 | 169 | Lytic enzyme |
|  | 1 | 23 | rIIB protector from prophage-induced early lysis |
| Photosynthesis-related Proteins |  |  |  |
| Electron transport | 2 | 1,407/699 | psbA/psbD: D1/D2 Protein |
| Electron transport | 1 | 224 | petE: Plastocyanin |
| Photoadaptation | 1 | 71 | speD: S - adenosylmethionine decarboxlase |
| Energy dissipation | 1 | 611 | HLIP: High light inducible protein |
| Phophate-related Proteins |  |  |  |
|  | 1 | 311 | pstS; periplasmic phosphate binding protein |
|  | 2 | 803 | phoH; phosphate starvation inducible protein |
| Carbon Metabolism |  |  |  |
|  | 1 | 600 | talC: Transaldolase |
| Other Functions |  |  |  |
| Nitrogen Fixation | 1 | 81 | nifU-like protein |
| Stress Response | 1 | 1072 | Small heat shock protein |
| Detoxification | 1 | 66 | Cu/Zn superoxide dismutase |
| Catalytic Activity | 1 | 588 | Dioxygenase |
| Synthesis of Biomolecules | 1 | 87 | Polyketide synthase |
| Energy Pathway | 1 | 434 | Carboxylesterase |
| Transposition | 1 | 23 | Transposase |
| Peptidase Activity | 1 | 20 | Serine peptidase family |
| Antibiotic Synthesis | 1 | 103 | Tryptophan halogenase (prnA) |
| Vitamin B12 biosynthesis | 1 | 833 | Cobalamin biosyntheis protein (cobS) |
| Small MutS related protein | 1 | 124 | Smr-like repressor |
| Cell Membrane Modification | 4 | 536 | Glycosyltransferases |
| **Total** | **127** | **61691** |  |
