# Supplementary material for: The Sorcerer II Global Ocean Sampling Expedition: Metagenomic Characterization of Viruses within Aquatic Microbial Samples
Source: PLoS One. 2008 Jan 23;3(1):e1456. doi: 10.1371/journal.pone.0001456 (PMC2186209; doi:10.1371/journal.pone.0001456)
Supplement: Table S3 — (0.06 MB DOC) [file pone.0001456.s012.doc]

| Table S3. Viral sequences belonging to GOS eukaryotic virus category. | | | |
| --- | --- | --- | --- |
| Functional Category | # of Clusters | # of Sequences | Protein Description (Putative) |
| DNA Replication |  |  |  |
|  | 4 | 499 | DNA polymerase |
|  | 1 | 223 | Proliferating cell nuclear antigen homolog |
|  | 1 | 255 | Replication factor |
| DNA Repair |  |  |  |
|  | 1 | 175 | CPD photolyase |
|  | 1 | 38 | DNA repair protein |
| Nucleotide Metabolism |  |  |  |
|  | 1 | 51 | Ribonucleoside diphosphate reductase |
|  | 1 | 311 | GDP -D-mannose dehydratase |
| Transcription |  |  |  |
|  | 1 | 25 | RNA helicase |
|  | 2 | 49 | DNA-directed RNA polymerase |
|  | 1 | 57 | mRNA guanylyltransferase |
|  | 3 | 352 | Transcription factor |
|  | 2 | 68 | TATA-box binding protein |
| Translation |  |  |  |
|  | 1 | 21 | Translation elongation factor |
|  | 1 | 125 | 33kDa translation peptide |
| Structural Proteins |  |  |  |
|  | 1 | 23 | Glucosamine synthetase |
|  | 1 | 300 | Core assembly protein |
|  | 1 | 1385 | Major capsid protein |
|  | 3 | 280 | Attachment protein |
|  | 2 | 118 | Envelope protein |
|  | 1 | 305 | Virion assembly ATPase |
|  | 2 | 65 | Capsid protein |
| Other Functions |  |  |  |
|  | 1 | 47 | Polyamine biosynthesis protein |
|  | 1 | 164 | Ribonuclease III - supression of RNA silencing during infection |
| DNA/Pantothenate Metabolism | 1 | 28 | HAL3 - like domain protein |
| Proteolysis | 1 | 32 | Viral cathepsin (cystein protease) |
| NAD Biosynthesis | 1 | 40 | Pyrazinamidase/Nicotinamidase |
| Protein Metabolism | 1 | 88 | Prolyl 4 - hydroxylase |
| Nucleotide Metabolism | 1 | 72 | Thymidine kinase |
| Protein-protein Interactions | 2 | 158 | Ankyrin repeat protein |
| Cell Membrane | 1 | 28 | Glycosyltransferase |
| **Total** | **42** | **5382** |  |
