# Supplementary material for: The Sorcerer II Global Ocean Sampling Expedition: Metagenomic Characterization of Viruses within Aquatic Microbial Samples
Source: PLoS One. 2008 Jan 23;3(1):e1456. doi: 10.1371/journal.pone.0001456 (PMC2186209; doi:10.1371/journal.pone.0001456)
Supplement: Table S4 — (0.04 MB DOC) [file pone.0001456.s013.doc]

| Table S4. Viral sequences belonging to GOS Mimivirus-like virus category. | | | |
| --- | --- | --- | --- |
| Functional Category | # of Clusters | # Sequences | Protein Description (Putative) |
| DNA Replication, Recombination and Repair |  |  |  |
|  | 1 | 27 | DNA topoisomerase I |
|  | 1 | 56 | DNA mismatch repair ATPase |
|  |  |  |  |
|  | 1 | 52 | Formamidopyrimidine DNA glycosylase |
|  | 1 | 72 | Cysteine methyltransferase |
|  | 1 | 30 | Resolvase |
|  | 1 | 148 | Endonuclease repair protein |
|  | 1 | 32 | SW1/SNF2 helicase |
| Transcription |  |  |  |
|  | 1 | 27 | A1L transcription factor |
| Amino Acid Metabolism |  |  |  |
|  | 1 | 38 | Asparagine synthase |
|  | 1 | 42 | Glutamine synthetase |
| Other Function |  |  |  |
| Catalytic Activity | 1 | 24 | HD phosphohydrolase |
| Catalytic Activity | 1 | 131 | Metal-dependent hydrolase |
| Collagen Crosslinking | 1 | 29 | Procollagen-lysine,2-oxoglutarate 5-dioxygenase |
| **Total** | **13** | **708** |  |
