# Supplementary material for: The Sorcerer II Global Ocean Sampling Expedition: Metagenomic Characterization of Viruses within Aquatic Microbial Samples
Source: PLoS One. 2008 Jan 23;3(1):e1456. doi: 10.1371/journal.pone.0001456 (PMC2186209; doi:10.1371/journal.pone.0001456)
Supplement: Table S5 — (0.04 MB DOC) [file pone.0001456.s014.doc]

| Table S5. Shared viral sequences belonging to both phage and eukaryotic virus categories. | | | | |
| --- | --- | --- | --- | --- |
| Functional Category | # of Clusters | # Sequences | Protein Description (Putative) | |
| DNA Replication |  |  |  | |
|  | 2 | 3048 | DNA helicase | |
|  | 1 | 173 | Type II DNA topoisomerase | |
|  | 1 | 2483 | DNA polymerase | |
|  | 2 | 400 | Exonuclease | |
|  | 1 | 1247 | Sliding clamp | |
| DNA Repair |  |  |  | |
|  | 2 | 100 | Deoxynucleoside triphosphate deaminase | |
|  | 1 | 74 | Global transactivator-like protein/DNA helicase | |
|  | 2 | 604 | DNA ligase | |
| DNA Modification |  |  |  | |
|  | 2 | 431 | DNA methylase | |
|  |  |  |  | |
| Nucleotide Metabolism |  |  |  | |
|  | 1 | 1959 | Aerobic NDP reductase, large subunit | |
|  | 1 | 107 | Thymidine kinase | |
|  | 2 | 1499 | Thymidylate synthase | |
| Protein-Protein Interactions |  |  |  | |
|  | 1 | 41 | Ankyrin repeat protein | |
| Structural Proteins |  |  |  | |
|  | 2 | 216 | Structural protein | |
| **Total** | **21** | **12,382** |  |  |
