# Supplementary material for: The Sorcerer II Global Ocean Sampling Expedition: Metagenomic Characterization of Viruses within Aquatic Microbial Samples
Source: PLoS One. 2008 Jan 23;3(1):e1456. doi: 10.1371/journal.pone.0001456 (PMC2186209; doi:10.1371/journal.pone.0001456)
Supplement: Table S6 — (0.09 MB DOC) [file pone.0001456.s015.doc]

| Table S6. Primer information for qPCR experiments. | | | |
| --- | --- | --- | --- |
| Primer name | Primer sequence | Tm^*^ | Amplicon length (bp) |
| talC_1F | TGTATCGGCAGAAGTTGTTGC | 58 | 69 |
| talC_1R | AGTCCTTCAAATTCCATTGCCT | 58 |  |
| talC_2F | TWCTWGCWCAAAAGCAG | 56 | 51 |
| talC_2R | CATCTTCCWACAAAWGGWGA | 55 |  |
| talC_3F | TTTGAYCAGATGTATGATCACA | 56 | 66 |
| talC_3R | TGCCCAATCTTTATCAAAGATTT | 56 |  |
| talC_4F | CAGGWGCAACTTATGTTTCACC | 57 | 58 |
| talC_4R | CCACCAAAAGAATTGTCATCAAC | 57 |  |
| talC_5F | CCATTTGTAGGWAGAGTTGATGA | 57 | 144 |
| talC_5R | AAATGCTCTRCCAACATCTCTTA | 56 |  |
| talC_6F | GCAGGTGCAAAGTATGTTTCA | 55 | 131 |
| talC_6R | TGCTGCAAGTATCTCTGTCTTTC | 55 |  |
| talC_7F | AGTATGGAAGTGTTGGTRAYC | 56 | 201 |
| talC_7R | TGCACCTGCTTTWGCWG | 57 |  |
| talC_8F | GGGAATCAGAGTAAACGTGACTT | 55 | 46 |
| talC_8R | CGCTTGAGCAGCACTAAAGA | 56 |  |
| talC_9F | CAACTTATAGTCATGAAAMTYTTTCTGG | 54 | 44 |
| talC_9R | TCRGGATCAGAGCAGTCCA | 55 |  |
| talC_10F | TCTCAGCAGCACAGGCAATAC | 58 | 46 |
| talC_10R | TATGCTGCTCCTGCCTTTGAT | 59 |  |
| talC_11F | CCCCGATGTCCATTATTMAC | 57 | 534 |
| talC_11R | GCGAGAGACRTGATGDACA | 57 |  |
| talC_12F | GCTAAAGCAGGTGCAACATATG | 56 | 41 |
| talC_12R | CGWCCAACAAAAGGAGAGAC | 59 |  |
| speD_1F | GCACTRGCAGAAAGTCACGTTT | 58 | 48 |
| speD_1R | CCCATTTTCTGGCCAAGTATGA | 60 |  |
| speD_2F | CTTGGCCTGAATTTGGTCG | 58 | 46 |
| speD_2R | CCGCAAGTGAAGAAATCTGC | 57 |  |
| speD_3F | TGAACCACAAGGWGTYACTGT | 56 | 103 |
| speD_3R | CCACAAGTATARACATCYACTGCTGC | 58 |  |
| speD_4F | TGCTTGCTGAGAGTCACATCA | 57 |  |
| speD_4R | TTTTCTGGCCAAGTGTGAATAC | 56 |  |
| pstS_1F | CGTTCCTCTTAAGGGTGACATT | 56 | 46 |
| pstS_1R | AACTGCAGCTCTTGACTTCTCA | 56 |  |
| pstS_2F | CGCATTGCTTCCTTTACTACTCAC | 58 | 82 |
| pstS_2R | TGAGGGGAAAGATGCACCTG | 60 |  |
| pstS_3F | CTCAAGAGCAAGCAGTACAAGTTG | 57 | 95 |
| pstS_3R | CAGAACGATGTGCCCAAGTT | 57 |  |
| pstS_4F | GAGTAAGGCCGAGGAACTTG | 55 | 46 |
| pstS_4R | CACACCGTCAGGAAGAGGAA | 57 |  |
| petE_1F | RCCWGGTGAAGAGTTTGA | 55 | 73 |
| petE_1R | CCCYTTATGAGGASCACACCA | 57 |  |
| petE_2F | AGAYCATCCCGAACTATCAC | 56 | 46 |
| petE_2R | CCCACCCATAAAAGCCARGTC | 58 |  |
| petE_3F | CACGAAATTRWGTGGTGGATG | 56 | 308 |
| petE_3R | CCWGCATGAGGWTCACA | 57 |  |
| petE_4F | TCCTGGWGAATCRCAAGA | 55 | 71 |
| petE_4R | CCTGATGAGGACCACASAAGAA | 55 |  |
| phoH_3F | GGTTTTCTACCRGGMAATCAA | 56 | 239 |
| phoH_3R | CCAACACGAGTSATGATAGAATC | 55 |  |
| phoH_4F | GATGGAGATCAACCMATTGG | 56 | 366 |
| phoH_4R | TCAGWWGGCATCTGAAACAT | 56 |  |
| phoH_5F | AAAAGAAAACTACCAAAGCAGCTGG | 60 | 46 |
| phoH_5R | TGTTTSGCAGTCATACCATTACC | 57 |  |
| phoH_6F | CATCATTCATTCGTGGAACAAC | 57 | 56 |
| phoH_6R | GWGACTCATCAACTAACACAATGC | 59 |  |
| psbD_F | CAACAACCAACAAAGAGGATGG | 58 | 46 |
| psbD_R | AAACCAGTCATCAAGGACATCA | 56 |  |
| * Melting temperature | | | |
