# Supplementary material for: The Sorcerer II Global Ocean Sampling Expedition: Metagenomic Characterization of Viruses within Aquatic Microbial Samples
Source: PLoS One. 2008 Jan 23;3(1):e1456. doi: 10.1371/journal.pone.0001456 (PMC2186209; doi:10.1371/journal.pone.0001456)
Supplement: Table S8 — (0.09 MB DOC) [file pone.0001456.s017.doc]

| Table S8. Description of tailed bacteriophage sequences contained on scaffolds ≥5kb. | | | | | | |
| --- | --- | --- | --- | --- | --- | --- |
|  |  |  |  |  |  |  |
| **Phage Family** | **Closest NR Homolog** | **Host** | **# Scaffolds** | **% of Total** | **# Phage Sequences** | **% of Total** |
|  |  |  |  |  |  |  |
| *Myoviridae* |  |  |  |  | 8,062 | 89.9 |
|  | Cyanophage S-PM2 | *Synechococcus sp.*  WH 7803 | 253 | 80.2 |  |  |
|  | Cyanophage P-SSM2 (T4-like) | *Prochlorococcus sp.* | 14 | 4.5 |  |  |
|  | Bacteriophage RM 378  (T4-like) | Thermophillic host | 3 | 1 |  |  |
|  | Bacteriophage KVP40 (T4-like) | *Vibrio sp.*- broad host range phage | 3 | 1 |  |  |
|  | Bacteriophage Aaphi 23 | Actinobacillus actinomycetemcomitans | 2 | 0.64 |  |  |
|  | Aeromonas phage 65 (T4-like) | *Aeromonas salmonicida* | 1 | 0.32 |  |  |
|  | Aeromonas phage 44RR2 .8t  (T4-like) | *Aeromonas salmonicida* | 1 | 0.32 |  |  |
|  | Family level only | N/A | 38 | 12.1 |  |  |
|  |  |  |  |  |  |  |
| *Podoviridae* |  |  |  |  | 514 | 5.7 |
|  | Pseudomonas aeruginosa phage PaP3 | *Pseudomonas aeruginosa* | 5 | 27.8 |  |  |
|  | Cyanophage P60 | *Synechococcus sp.* WH 7803 | 3 | 16.7 |  |  |
|  | Burkholderia cepacia phage Bcep22 | *Burkholderia cepacia* | 1 | 5.6 |  |  |
|  | Vibriophage VP5 | *Vibrio cholerae* | 1 | 5.6 |  |  |
|  | Bordetella phage BIP-1 | *Bordetella bronchiseptica* | 1 | 5.6 |  |  |
|  | Bacteriophage Mx8 (P22-like) | Myxococcus xanthus strain DK883 | 1 | 5.6 |  |  |
|  | Enterobacteriophage epsilon 15  (P22-like) | *Salmonella enterica* | 1 | 5.6 |  |  |
|  | Yersinia pestis phage phi A1122  (T7-like) | *Yersinia pestis* | 1 | 5.6 |  |  |
|  | Family level only | N/A | 4 | 22.2 |  |  |
|  |  |  |  |  |  |  |
| *Siphoviridae* |  |  |  |  | 388 | 4.3 |
|  | Bacteriophage P27  (lambda like) | *Escherichia coli* | 1 | 12.5 |  |  |
|  | Bacteriophage phi E125  (lambda like) | *Burkholderia thailandensis* | 1 | 12.5 |  |  |
|  | Mycobacteriophage Rosebush | *Mycobacterium smegmatis* | 1 | 12.5 |  |  |
|  | Bacteriophage B3 | *Pseudomonas aeruginosa* PAO1 | 1 | 12.5 |  |  |
|  | Bacteriophage PY54 | *Yersinia enterocolitica* | 1 | 12.5 |  |  |
|  | Enterobacteriophage T5 | *Escherichia coli* | 1 | 12.5 |  |  |
|  | Mycobacteriophage Che8 | *Mycobacterium smegmatis* | 1 | 12.5 |  |  |
|  | Bacteriophage lambda | *Escherichia coli* | 1 | 12.5 |  |  |
| **Total** |  |  | **341** |  | **8,964** | **100** |
