# Supplementary material for: The Sorcerer II Global Ocean Sampling Expedition: Metagenomic Characterization of Viruses within Aquatic Microbial Samples
Source: PLoS One. 2008 Jan 23;3(1):e1456. doi: 10.1371/journal.pone.0001456 (PMC2186209; doi:10.1371/journal.pone.0001456)
Supplement: Table S9 — (0.05 MB DOC) [file pone.0001456.s018.doc]

| Table S9. Description of eukaryotic virus sequences contained on scaffolds ≥5kb. | | | | | | | |
| --- | --- | --- | --- | --- | --- | --- | --- |
| **Viral family** | **Viral genus** | **Closest NR homolog** | **Host** | **# Scaffolds** | **% Total** | **# Viral sequences** | **% Total** |
| *Phycodnaviridae* |  |  |  |  |  | 2073 | 76.9 |
|  | Chlorovirus | *Paramecium bursaria* Chlorella virus 1 | *Chlorella* strain NC64A | 31 | 44.3 |  |  |
|  | Chlorovirus | No Information | No Information | 12 | 17.1 |  |  |
|  | Phaeovirus | *Ectocarpus siliculosus* virus | *Ectocarpus siliculosus* | 2 | 2.9 |  |  |
|  | Family level only | N/A | N/A | 25 | 35.7 |  |  |
| Mimivirus |  |  |  |  |  | 535 | 19.8 |
|  | N/A | *Acanthamoeba polyphaga* mimivirus | *Acanthamoeba polyphaga* | 5 | 100 |  |  |
| *Herpesviridae* |  |  |  |  |  | 41 | 1.5 |
|  | Rhadinovirus | No Information | N/A | 2 | 66.7 |  |  |
|  | Varicellovirus | No Information | N/A | 1 | 33.3 |  |  |
| *Poxviridae* |  |  |  |  |  | 29 | 1.1 |
|  | No Information | No Information | N/A | 2 | 100 |  |  |
| *Iridoviridae* |  |  |  |  |  | 18 | 0.7 |
|  | Iridovirus | Invertebrate iridescent virus | Invertebrates | 1 | 100 |  |  |
| **Total** |  |  |  | **81** | N/A | **2696** | **100** |
