# Supplementary material for: The Sorcerer II Global Ocean Sampling Expedition: Metagenomic Characterization of Viruses within Aquatic Microbial Samples
Source: PLoS One. 2008 Jan 23;3(1):e1456. doi: 10.1371/journal.pone.0001456 (PMC2186209; doi:10.1371/journal.pone.0001456)
Supplement: Table S10 — (0.04 MB DOC) [file pone.0001456.s019.doc]

| Table S10. BLAST-based taxonomic details for GOS viral sequences contained on scaffolds ≥5kb. | | | | | |
| --- | --- | --- | --- | --- | --- |
| **% Bases assigned to given tax. level** | **% All bases assigned to a tax. ID** | **Scaffold ID** | **Scaffold length (kb)** | **% All bases unknown** | **Taxonomic ID^a^** |
|  |  |  |  |  |  |
| 100 | 34.2 | 1096627390297 | 31202 | 65.7 | Myoviridae |
| 59.5 | 48.2 | 1096627384948 | 29474 | 19 | Myoviridae |
| 100 | 94.2 | 1096627381107 | 26427 | 5.7 | Myoviridae |
| 100 | 86.8 | 1096627379460 | 26267 | 13.1 | Myoviridae |
| 100 | 62.7 | 1096627390447 | 24486 | 37.2 | Myoviridae |
| 100 | 55.5 | 1096627220969 | 23532 | 44.4 | Myoviridae |
| 84.5 | 33.7 | 1096627389713 | 22001 | 60 | Myoviridae |
| 81.4 | 17.2 | 1096627004926 | 20583 | 78.8 | Myoviridae |
| 100 | 11.7 | 1096627257231 | 10779 | 88.2 | T4-like viruses |
| ^a^ *Myoviridae* and T4-like viruses represent examples of possible taxomonic identifications at varying levels of resolution. | | | | | |
